# Mussel‐Inspired Self‐Assembly of PtO_4_ Atomic Catalysts for Interfacial Synergistic Hydrogen Evolution

**DOI:** 10.1002/advs.202507807

**Published:** 2025-06-29

**Authors:** Yeo Hoon Yoon, Karthikeyan Jeyakumar, Gang San Lee, Jayaraman Balamurugan, Suchithra Padmajan Sasikala, Chan Woo Lee, Colin Wing‐Lok Cheng, Jun Beom Kim, Haeshin Lee, Sang Ouk Kim

**Affiliations:** ^1^ National Creative Research Initiative Center for Multi‐Dimensional Directed Nanoscale Assembly Department of Materials Science and Engineering KAIST Daejeon 34141 Republic of Korea; ^2^ Department of Physics National Institute of Technology Durgapur West Bengal 713209 India; ^3^ Department of Chemistry Korea Advanced Institute of Science and Technology Daejeon 34141 Republic of Korea

**Keywords:** hydrogen evolution reaction, interfacial synergy effects, mussel‐inspired, pyrolysis‐free, single atom catalysts

## Abstract

Molecular self‐assembly strategy for Pt‐O electrocatalysts is presented, achieved by facile solution processing of dopamine monomers. This mussel‐inspired strategy ensures a highly defined PtO_4_ atomic structure with uniform monolayer deposition across various 2D nanomaterials. By leveraging the intimate interplay between PtO_4_ sites and 2D substrates, while maintaining a consistent coordination environment, underlying mechanism for enhanced kinetics in interfacial synergistic hydrogen evolution reaction is systematically elucidated. Notably, the self‐assembled PtO_4_ site exhibits up to a 30‐fold enhancement of mass activity compared to commercial Pt/C catalysts, contingent upon the choice of substrate material. Theoretical investigation illustrates facilitated electron transfer, optimized energy barriers, and additional reaction pathways resulting from the synergistic interplay between PtO_4_ and Ti_3_C_2_O_2_ MXene. This straightforward, energy‐efficient, and highly reliable scheme for atomic‐level catalysts prospects a valuable platform toward sub‐molecular level engineering of tailored electronic structures and properties.

## Graphical abstract

Mussel‐inspired strategy for self‐assembling PtO_4_ atomic catalysts on different 2D substrates is presented. Notably, the self‐assembled PtO_4_ site exhibits up to a 30‐fold enhancement of mass activity compared to commercial Pt/C catalysts, contingent upon the choice of substrate material. This work highlights the critical role of non‐covalent interfacial engineering in catalytic performance.

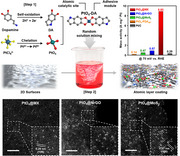

## 1 Introduction

The hydrogen evolution reaction (HER) is a vital element for the green energy ecosystem with high energy density as well as inherent zero‐emission characteristics.^[^ ^1^, ^2^ ^]^ Platinum (Pt)‐based catalysts have been principally utilized to activate this reaction scheme, taking advantage of the optimal characteristics for hydrogen binding.^[^ ^3^, ^4^ ^]^ Unfortunately, the intrinsic high cost arising from the scarcity of Pt has been a longstanding bottleneck for the practical utilization. Since the discovery of Fe‐N_4_ and other single‐atom catalysts (SACs),^[^ ^5^, ^6^, ^7^, ^8^, ^9^ ^]^ Pt‐based atomic catalytic units have attracted a great deal of research attentions, particularly owing to the maximal atomic utilization and favorable electronic structure.^[^ ^10^, ^11^, ^12^ ^]^

Apart from the delicate metal‐support electronic interactions that modulate the *d*‐band occupation near the Fermi level,^[^ ^13^, ^14^ ^]^ various strategies have been developed thus far to promote the catalytic activity of Pt atomic sites for HER.^[^ ^15^, ^16^ ^]^ For instance, recent studies have discovered the excellent catalytic activity of Pt─O bonding in acidic HER, even surpassing that of Pt metals.^[^ ^17^, ^18^, ^19^ ^]^ The oxygen (O) atom not only serves as an additional active site with an optimal proton binding energy but also kinetically facilitates the hydrogen desorption.^[^ ^20^ ^]^ In addition, Pt─O─Pt atomic clusters stabilized on isolated cobalt single atoms (Pt‐ACs/CoNC) were developed as an efficient HER electrocatalyst, in which the effective interaction at the Pt─O─Co interface induces favorable charge density redistribution at both Pt and O atoms, kinetically facilitating the hydrogen evolution.^[^ ^21^ ^]^ These studies underscore the significance of electron redistribution at Pt─O active sites for maximizing catalytic activity, while also suggesting an increased interest in developing interfacial strategies to facilitate and optimize the electron density more effectively. To further advance this promising aspect, more precise and uniform model structures that enable in‐depth mechanistic studies are highly demanded. Unfortunately, the uncontrolled reactivity of O atoms at high‐energy conditions, involving high‐temperature fabrication or electrochemical deposition, poses significant challenges in precisely modelling the Pt─O atomic units.

In this work, inspired by the excellent adhesive nature and facile chelation reaction of dopamine,^[^ ^22^, ^23^ ^]^ we successfully assembled well‐defined monodisperse PtO_4_ atomic catalytic sites on various 2D substrates, including Graphene, MXene, and Transition metal dichalcogenides (TMDs), via an ambient random solution mixing process. Dopamine is a natural substance, derived from mussel adhesive proteins, whose low energy barrier for chelation reaction not only effectively prevents the aggregation of metal atoms,^[^ ^24^, ^25^, ^26^ ^]^ but also ensures the uniform PtO_4_ atomic configuration. The opposite charge states at 2D substrates lead to the electrostatic self‐assembly, uniformly distributing the monolayered PtO_4_ atomic units on 2D surfaces. Importantly, this mussel‐inspired approach offers an idealized model system for investigating the PtO_4_ active sites, while integrating the different types of substrate materials. This facile yet highly effective interfacial interaction enables the precise modulation of electron redistribution, thereby enhancing the HER efficiency. Remarkably, PtO_4_ self‐assembled at MXene surfaces (PtO_4_@MX) exhibits 63 times greater mass activity than the counterpart without MXene (PtO_4_‐PDA_NP_), highlighting the profound impact of interfacial synergistic effects with underlying substrates. Density functional theory (DFT) revealed the detailed electrocatalytic mechanism, further elucidating the role of interfacial synergistic effects in terms of facile electron redistribution. Notably, the calculated energy barrier for the rate‐determining step in PtO_4_@MX is 0.26 eV, significantly lower than 0.66 eV of Pt (111). This study proposes an idealized design principle for catalytic systems with subtle synergistic cooperation between atomic configuration and supporting substrate materials.

## 2 Results and Discussion

### 2.1 Preparation and Characterization of PtO_4_@MX

**Figure 1:**
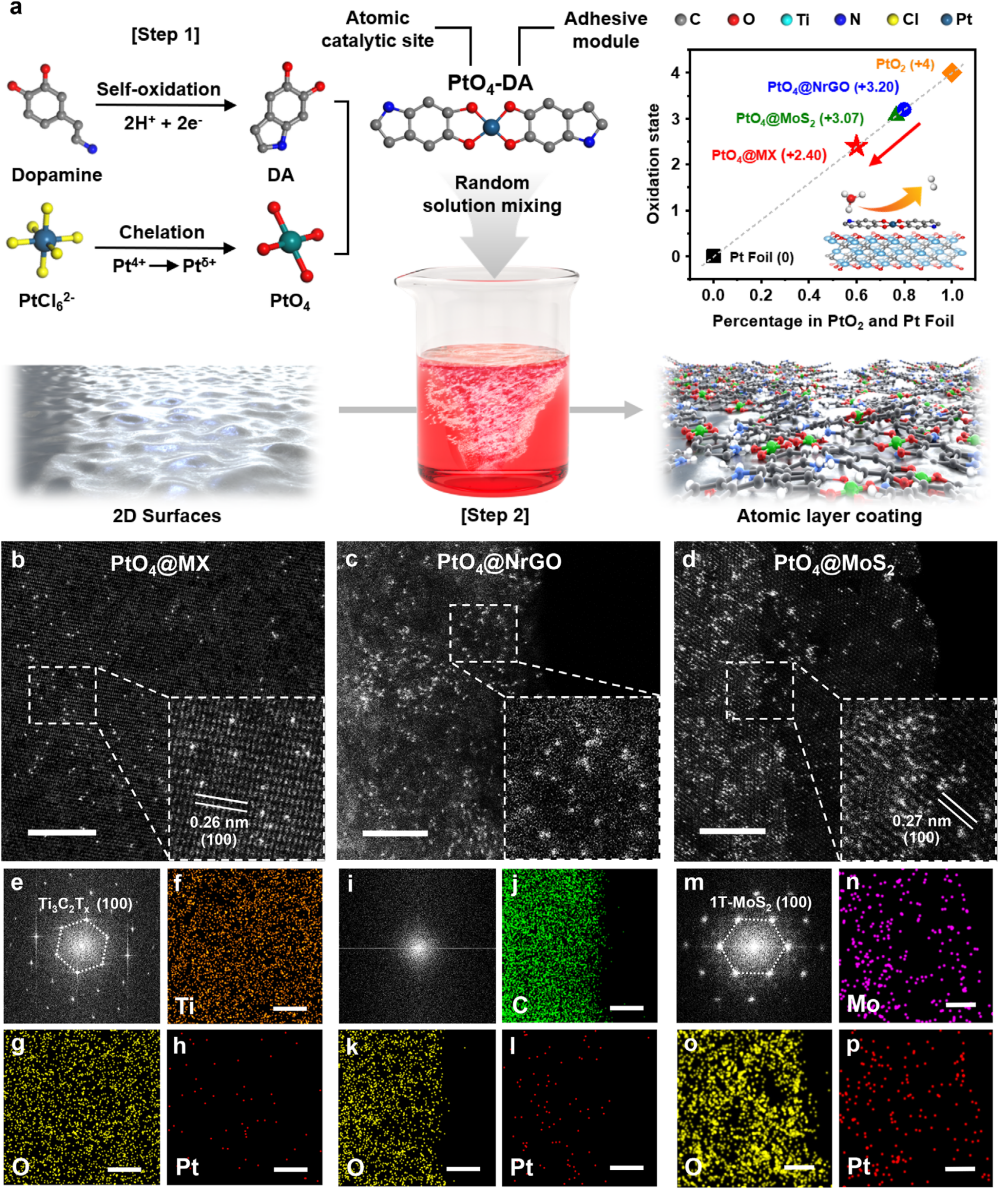
Preparation of PtO_4_ atomic catalysts. a) Schematic illustration of the two‐step synthesis strategy for mussel‐inspired PtO_4_ atomic sites self‐assembled on various 2D nanomaterials with distinct electron redistributions. b–d) HAADF‐STEM images of PtO_4_@MX, PtO_4_ @NrGO, and PtO_4_ @MoS_2_ with magnified inset images and measured lattice fringes. e,i,m) Corresponding FFT patterns with specific lattice planes. f–h) Corresponding elemental mapping of Ti, O, and Pt in the PtO_4_@MX. j,k,l) Corresponding elemental mapping of C, O, and Pt in the PtO_4_@NrGO. n–p) Corresponding elemental mapping of Mo, O, and Pt in the PtO_4_@MoS_2_. All the scale bars are 500 nm.

Based on the straightforward room‐temperature solution processing of adhesive dopamine precursors, uniform PtO_4_ atomic sites are successfully incorporated on various 2D surfaces (Figure S1, Supporting Information). The typical protocol for PtO_4_@MX consists of two distinct stages: atomically precise synthesis of PtO_4_ active sites via dopamine chelation and subsequent electrostatic stabilization on 2D nanomaterials (**Figure** **1**a). In our proposed mechanism of dopamine self‐oxidation illustrated in Figure S2 (Supporting Information), ammonium cations can effectively suppress the covalent polymerization via cation‐π interaction, while allowing the cyclization of amine chains (See Figure S2, Supporting Information for synthetic details).^[^ ^27^ ^]^ Attributed to the reductive environment,^[^ ^28^ ^]^ the Pt‐catechol coordination bonding can be efficiently stabilized into Pt─O covalent bonds, yielding the Pt‐chelated dopamine (PtO_4_‐DA) in low‐energy states. By contrast, the synthesis of PtO_4_‐DA under alkaline conditions readily leads to the formation of polydopamine nanoparticles (PtO_4_‐PDA_NP_) as shown in Figures S3 and S4 (Supporting Information).

**Figure 2:**
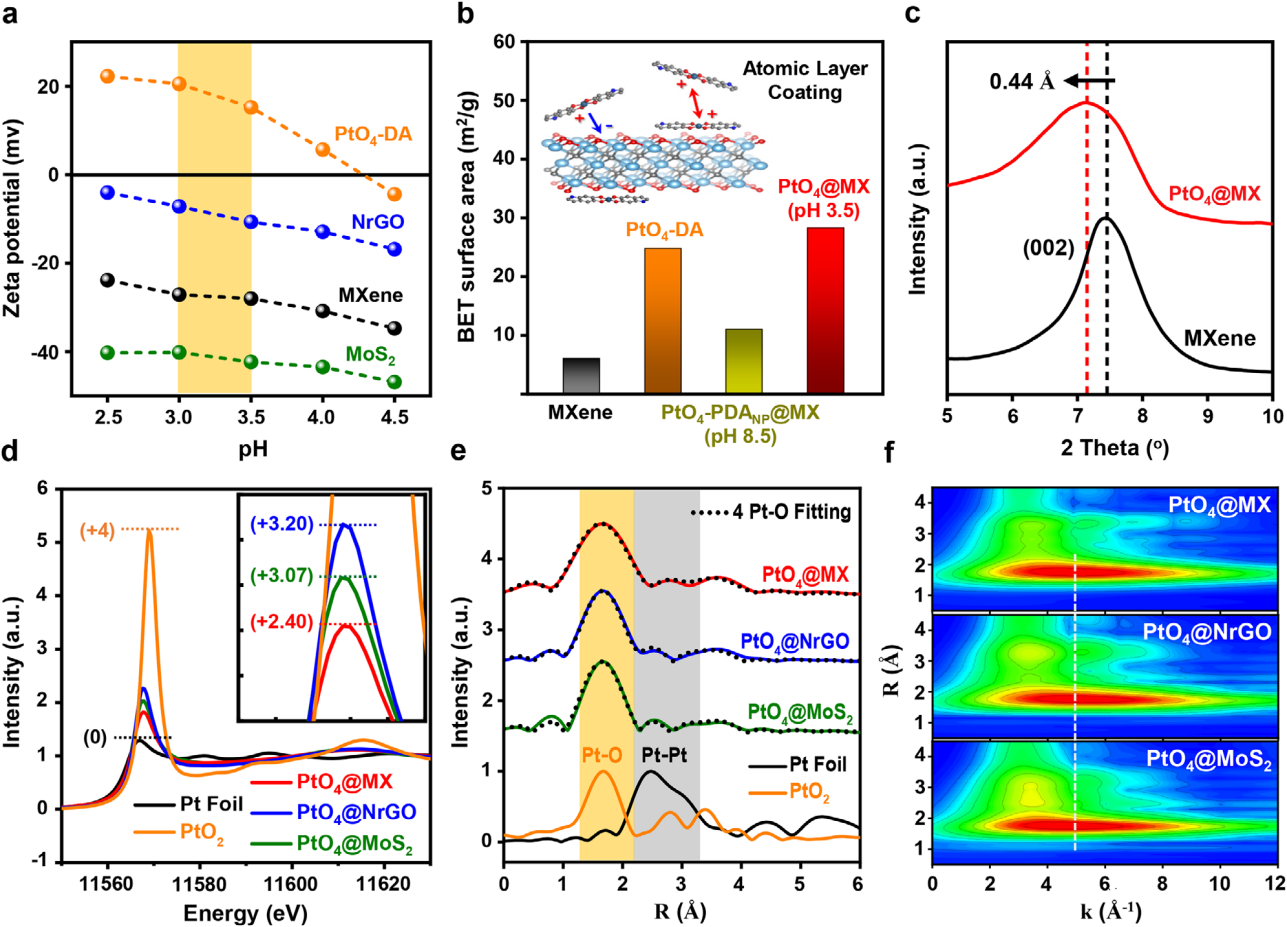
Structure characterizations of PtO_4_ atomic catalysts. a) Zeta potential measurements of PtO_4_‐DA, NrGO, MXene, and MoS_2_. b) BET surface areas for pristine MXene, PtO_4_‐DA, PtO_4_‐PDA_NP_@MX, and PtO_4_@MX with the schematic illustration of electrostatic self‐assembly. c) XRD θ‐2θ spectra of pristine MXene and PtO_4_@MX. d) XANES spectra at the Pt‐L_3_ edge of PtO_4_@MX, PtO_4_@NrGO, PtO_4_@MoS_2_, PtO_2_, and reference Pt foil with the calculated oxidation states. e) Corresponding FT‐EXAFS curves and fitting curves with 4 Pt‐O pathways. f) Corresponding WT‐EXAFS plots.

The electrostatic substrate deposition was also conducted in an acidic condition for the uniform dispersion of PtO_4_‐DA on Ti_3_C_2_T_x_ MXene surfaces. Owing to the opposite surface charge between the as‐assembled PtO_4_‐DA and MXene in the acidic pH condition (**Figure** **2**a), the electrostatic attraction effectively facilitates the self‐assembly of PtO_4_@MX, whereas the repulsive force between PtO_4_‐DA prevents aggregation. The importance of an acidic environment is further verified by the differences in Brunauer–Emmett–Teller (BET) surface areas of each sample derived from their nitrogen adsorption/desorption isotherms (Figure S5, Supporting Information). MXene exhibits a BET surface area of 5.90 m^2^ g^−1^, whereas PtO_4_@MX demonstrates a significantly higher value of 28.2 m^2^ g^−1^, surpassing the 24.7 m^2^ g^−1^ of PtO_4_‐DA (Figure 2b). This enhancement is attributed to the well‐dispersed PtO_4_‐DA on the MXene surface. Consequently, PtO_4_‐DA can achieve an exceptional atomic‐scale dispersion on the substrates at pH 3.5 (Figure S6, Supporting Information) with a negligible change in BET surface area, whereas PtO_4_‐DA strongly aggregates into clusters at pH 8.5 (Figure S7, Supporting Information). This underscores the critical role of pH conditions as a key parameter for our electrostatic self‐assembly.^[^ ^29^, ^30^, ^31^ ^]^

The structure evolution of PtO_4_‐DA is studied by X‐ray photoelectron spectroscopy (XPS) and Fourier transform Infrared (FT‐IR) spectroscopy. The XPS O 1s spectrum of PtO_4_‐DA only shows the negative shift (−0.4 eV) in the deconvoluted C─O peak, attributed to the formation of C─O─Pt bonds from the initial C─O─H bonds, while inducing a higher electron density (Figure S8, Supporting Information). The intensity of the C═O deconvoluted peak also decreased with the Pt─O bond formation. No significant difference is observed except for the minor shift of the deconvoluted C─O peak in XPS C 1s and N 1s spectra, supporting the selective chelation reaction between catechol groups and Pt ions (Figure S9, Supporting Information). Chloride (Cl^−^) from the initial PtCl_6_^2−^ precursor was completely removed upon the nucleophilic reaction with catechol groups, as confirmed by the absence of XPS Cl 2p signal (Figure S10, Supporting Information). In FT‐IR spectra, the C─O─H conformation in 1502 shifted to 1467 cm^−1^, indicating the presence of heavier Pt atoms instead of hydrogen atoms (Figure S11, Supporting Information). The detailed electronic state and coordination environment of Pt atoms are verified by X‐ray absorption near‐edge structure (XANES), and Fourier transform extended X‐ray fine structure (FT‐EXAFS) of PtO_4_‐DA, PtO_2_, and reference Pt foil (Figure S12a, Supporting Information). The valence state of Pt can be quantitatively investigated by calculating the highest derivative points at the pre‐edge region of each sample with the relative contribution based on a reference Pt foil and PtO_2_ (Figure S12b, Supporting Information).^[^ ^13^, ^21^, ^32^ ^]^ The calculated oxidation state of PtO_4_‐DA is + 3.00, noticeably higher than Pt foil (0) but lower than PtO_2_ (+4). The white‐line intensity also followed the same tendency, indicating the high charge loss of the Pt single atom. FT‐EXAFS results of Pt L_3_‐edge in PtO_4_‐DA further confirmed the single atomic structure with the dominant Pt─O peak at 1.65 Å, which shares a similar peak position to the representative Pt‐O coordination environment in PtO_2_ (Figure S12c, Supporting Information). The absence of a typical broad Pt‐Pt peak appearing at 2.4 Å and Pt─O─Pt lattice peak at 2.7 Å further illustrates the unique configuration of PtO_4_‐DA. Furthermore, the first‐shell EXAFS fitting precisely confirms the high yield of uniform PtO_4_ atomic sites (Figure S12d, Supporting Information).

To investigate the detailed structure evolution of PtO_4_@MX, Pt nanoparticles on MXene (Pt@MX) catalyst were prepared as a reference, by using a simple wet‐impregnation method with reducing agents (Figure S13, Supporting Information). As shown in the high‐resolution XPS Pt 4f spectra (Figure S14, Supporting Information), Pt@MX exhibits dominant metallic Pt peaks, as Pt atoms tend to agglomerate into metal nanoclusters under reductive environments. Conversely, PtO_4_@MX only shows major 2+ oxidation state peaks at 75.5 and 72.2 eV for the isolated Pt atoms. DFT simulations are conducted to compare the binding energies for Pt─O covalent bonding and Pt─Pt metallic bonding (Figure S15, Supporting Information). The calculated energy for Pt‐O chelation is significantly lower than that for Pt metal nucleation, even at high Pt concentrations, well‐supporting the spontaneous formation of PtO_4_ atomic sites. X‐ray diffraction (XRD) analysis verifies the absence of the Pt (111) metal peak in PtO_4_@MX (Figure S16, Supporting Information). Furthermore, no structural deformation is observed between PtO_4_@MX and pristine MXene, suggesting a negligible effect from PtO_4_‐DA species in the structure of MXene. The slight downfield shift of (002) from 7.44° to 7.17° can be explained by the increase in MXene *d*‐spacing along with the atomic deposition of PtO_4_‐DA (Figure 2c), which is only 0.44 Å for the uniform monolayer coating at the surfaces.^[^ ^33^ ^]^

### 2.2 Identical PtO_4_ Atomic Sites on Various 2D Substrates

This mussel‐inspired strategy can be employed to various substrates such as Ti_3_C_2_T_x_ MXene, N‐doped reduced Graphene Oxide (NrGO), and Molybdenum Disulfide (MoS_2_) owing to the generic adhesion feature of dopamine.^[^ ^22^ ^]^ Those pristine 2D nanomaterials were prepared via typical top‐down liquid exfoliation methods (See Experimental Section for synthetic details & Figures S17–S19, Supporting Information for characterizations).^[^ ^34^, ^35^ ^]^ Under the identical preparation conditions with PtO_4_@MX, we successfully self‐assembled PtO_4_@NrGO and PtO_4_@MoS_2_ (Figure S20, Supporting Information). The electrostatic attraction derived from the opposite charge states between dopamine and 2D nanomaterials efficiently prevented the aggregation of PtO_4_‐DA, resulting in the uniform atomic‐scale stabilization on various surfaces (Figure 2a). The zeta‐potential measurements revealed that the 2D nanomaterials became neutralized after the electrostatic self‐assembly with the oppositely charged PtO_4_‐DA (Figure S21, Supporting Information). The XRD patterns of PtO_4_@NrGO and PtO_4_@MoS_2_ verified the absence of the Pt (111) peak and showed negligible structural changes compared to pristine substrates. No evidence of the broad amorphous peak typically associated with PDA_NP_ was detected (Figure S22, Supporting Information).

DFT simulations are conducted to calculate the adsorption energy of PtO_4_‐DA on each substrate to clarify the primary mechanism for atomic dispersion (Figure S23, Supporting Information). The obtained high adsorption energies corroborate the strong driving force for electrostatic self‐assembly, well‐supporting the uniform dispersion of Pt single atoms across different substrates, including Ti_3_C_2_O_2_, pristine graphene, and 2H‐MoS_2_. The as‐prepared PtO_4_@MX, PtO_4_@NrGO, and PtO_4_@MoS_2_ were characterized using aberration‐corrected high‐angle annular dark‐field scanning transmission electron microscopy (HAADF‐STEM). High‐magnified HAADF‐STEM images in Figure 1b–d reveal distinct bright spots corresponding to high atomic number elements, unequivocally confirming the successful incorporation of uniformly distributed Pt single atoms across the entire substrates. The magnified inset images and corresponding fast Fourier transform (FFT) patterns in Figure 1e,i,m further delineate the detailed substrate structures. The MXene substrates exhibit distinct (100) crystallographic planes with the lattice fringes of 0.26 nm,^[^ ^36^ ^]^ while MoS_2_ predominantly comprises the 1T phase structure characterized by the (100) basal planes with the lattice fringes of 0.27 nm.^[^ ^37^ ^]^ Notably, no amorphous rings indicative of dopamine cluster formation are observed. Elemental mapping images in Figure 1f,j,n highlight the core elements of each substrate, including Titanium (Ti), Carbon (C), and Molybdenum (Mo). Concurrently, Figure 1g,k,o confirms the presence of O, attributed to the surface functional groups on the substrates and PtO_4_‐DA. Moreover, Figure 1h,l,p provides compelling evidence for the presence of Pt atoms, further substantiating the successful synthesis of PtO_4_ atomic sites.

To investigate the detailed valence states and atomic environments of PtO_4_@MX, PtO_4_@NrGO, and PtO_4_@MoS_2_, XANES and FT‐EXAFS were performed. As shown in Figure 2d, the XANES spectra reveal the distinct features with varying intensities of white‐line peaks corresponding to the transition of core electrons from Pt 2p orbitals to 5d states, serving as an indicator of the Pt 5d orbital occupancy. Hence, the lower peak intensity of PtO_4_@MX implies higher 5*d*‐band occupancy compared to PtO_4_@NrGO and PtO_4_@MoS_2_, indicating stronger interfacial interaction between PtO_4_ atomic sites and MXene substrates. The oxidation states quantitatively calculated from the pre‐edge line confirm the higher electron density of Pt in PtO_4_@MX (+2.40) compared to PtO_4_@NrGO (+3.20) and PtO_4_@MoS_2_ (+3.07), which follows the same tendency with the white‐line area intensities (Figure S24, Supporting Information). The XPS Pt 4f spectra in Figure S25 (Supporting Information) also show the negative shift of PtO_4_@MX, consistent with the XANES results indicating a higher electron density in Pt. The positive shift of XPS Ti 2p spectra and negative shift of XPS N 1s spectra in PtO_4_@MX further support these tendencies and suggest the facile electron transfer from MXene to PtO_4_‐DA (Figure S26, Supporting Information). Interestingly, this change is not observed in other substrates, as demonstrated by the XPS Mo 3d and N 1s spectra of MoS_2_ in Figure S27 (Supporting Information). Raman spectroscopy, a complementary surface‐sensitive analytical technique, revealed that PtO_4_@MX was the only substrate exhibiting a redshift, indicative of reduced electron density at the Ti sites resulting from surface‐mediated electron transfer (Figure S28, Supporting Information).

Despite the pronounced differences in electronic states, the first‐shell EXAFS fitting (Figure 2e) precisely verified the identical PtO_4_ atomic environments in PtO_4_@MX, PtO_4_@NrGO, and PtO_4_@MoS_2_, inferring that the interfacial charge transfer does not affect the atomic structure of the PtO_4_ active site itself. The fitting results confirm that all samples share the same Pt─O coordination pathway of PtO_4_‐DA with the same coordination number (CN) of 4.0, irrespective of the substrate type (Figure S29, Supporting Information). The fitting process employed a DFT‐optimized structure of planar PtO_4_ atomic sites, and the low R‐factor values further validate the accuracy of the fitting results (Table S1, Supporting Information). The wavelet transform (WT), a powerful technique capable of simultaneously reflecting structural information in both k and R spaces, is employed to further substantiate the experimental results. In the WT contour plot, PtO_4_@MX, PtO_4_@NrGO, and PtO_4_@MoS_2_ exhibit the intensity maximum at the identical values in both R and k spaces, verifying a consistent Pt coordination environment across all the samples (Figure 2f). This high uniformity starkly contrasts with the WT contour plots of reference Pt foil and PtO_2_ (Figure S30, Supporting Information), which distinctly highlight the presence of Pt─Pt and Pt─O─Pt configurations. Taken together, compared to other SAC studies, the PtO_4_ atomic catalytic sites fabricated through molecular self‐assembly without external energy input exhibit an exceptionally high precision in its single atomic structure across different types of 2D substrates, free from random bonding or unexpected deformation. Furthermore, it is noteworthy that the electronic state of the Pt atomic sites exhibited variations depending on the species of self‐assembled substrates.

### 2.3 HER Performance of PtO_4_@MX

**Figure 3:**
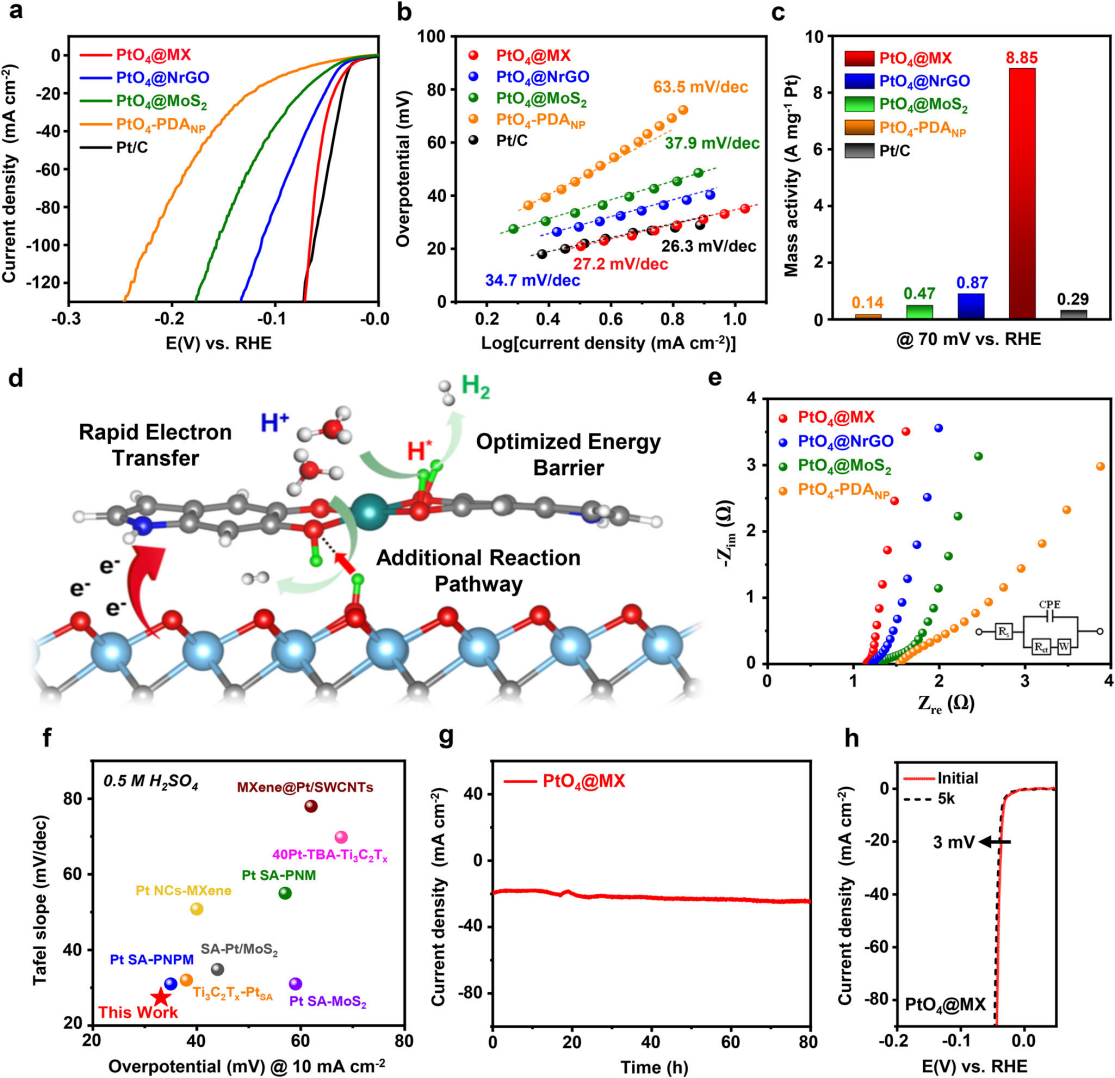
HER performance. a) HER polarization curves of PtO_4_@MX, PtO_4_@NrGO, PtO_4_@MoS_2_, PtO_4_‐PDA_NP_, and commercial Pt/C catalysts in 0.5 m H_2_SO_4_ solution. b) The Tafel slope originated from the LSV curves. (c) Calculated mass activity of the catalysts at the overpotential of 70 mV. d) Schematic illustration of interfacial synergistic hydrogen evolution reaction in PtO_4_@MX. e) EIS Nyquist plots of PtO_4_@MX, PtO_4_@NrGO, PtO_4_@MoS_2_, and PtO_4_‐PDA_NP_ with the equivalent circuit. f) Comparison plot of Tafel slope and overpotential at 10 mA cm^−2^ for various Pt‐based catalysts on 2D nanomaterials. g) Chronoamperometry curve of PtO_4_@MX obtained at the overpotential of −40 mV for 80 h. h) LSV curves of PtO_4_@MX before and after 5000 LSV cycles at a scan rate of 5 mV s^−1^. All the measurements were conducted in the identical conditions.

The HER catalytic activity of PtO_4_@MX was evaluated using a three‐electrode system in 0.5 m H_2_SO_4_ solution at ambient temperature. All the synthesized catalysts were uniformly drop‐cast onto carbon paper (CP) under identical conditions (Detailed procedures in the Experimental Section).^[^ ^10^ ^]^ As depicted in **Figure** **3**a, the HER polarization curves of PtO_4_ active sites exhibited substantial variations depending on the nature of substrates. Intriguingly, PtO_4_@MX displayed an exceptionally low Tafel slope of 27.2 mV dec^−1^ and the minimal overpotential of 33.1 mV to achieve a current density of 10 mA cm^−2^, comparable to that of the 40 wt.% commercial Pt/C catalyst (Figure 3b). Such a high performance is even more pronounced when considering mass activity, an essential metric for objectively assessing HER performance. Specifically, PtO_4_@MX achieved a remarkably high mass activity of 8.85 A mg^−1^, exceeding those of PtO_4_@NrGO, PtO_4_@MoS_2_, and PtO_4_‐PDA_NP_ by factors of 10, 18, and 63, respectively (Figure 3c). The precise Pt loading in each sample was measured by inductively coupled plasma‐optical emission spectroscopy (ICP‐OES) (Table S2, Supporting Information).

The substantial enhancement in mass activity underscores the synergistic effect at the hetero‐interface between the MXene substrate and PtO_4_ active sites, which facilitates the Volmer–Tafel mechanism for HER under acidic conditions.^[^ ^21^, ^38^ ^]^ Importantly, the observed catalytic enhancement is not attributed to the intrinsic activity of 2D substrates. Pristine MXene exhibited sluggish Tafel reaction and significantly higher overpotentials compared to NrGO and MoS_2_, primarily due to its excessively high proton binding energy (Figure S31, Supporting Information). Hence, the superior performance is predominantly governed by the PtO_4_ active sites, with their efficacy being highly dependent on their interaction with the underlying substrates. The markedly lower electrocatalytic activity of PtO_4_‐PDA_NP_ and PtO_4_‐PDA_NP_@MX in Figure S32 (Supporting Information)can be attributed to the less efficient charge transport through the low‐conductive PDA_NP_. The inferior catalytic performance of Pt@MX further indicates that Pt‐O active sites, rather than conventional Pt─Pt active sites, are more favorable for acidic HER. As illustrated in the comparison plot of Figure 3f, our PtO_4_@MX catalyst exhibits competitive overpotential and Tafel slope values relative to the most of the previously reported Pt catalysts derived from similar 2D platforms, validating the potential of this self‐assembled catalytic system (Table S3, Supporting Information).

Figure 3e presents the Nyquist plots of PtO_4_@MX, PtO_4_@NrGO, PtO_4_@MoS_2_, and PtO_4_‐PDA_NP_ that display significant differences in the charge transfer resistance (R_ct_), which is a critical parameter for evaluating the underlying synergy mechanism.^[^ ^39^ ^]^ Given the identical electrochemical setups and conditions, the reduced semicircle diameter of PtO_4_@MX indicates the synergistic interactions between MXene and PtO_4_ with the rapid charge transfer and facilitated hydrogen evolution kinetics at the active sites (Table S4, Supporting Information). To further elucidate the underlying catalytic mechanisms, the electrochemical double‐layer capacitance (C_dl_) was measured (Figure S33, Supporting Information) to estimate the electrochemical surface area (ECSA). The cyclic voltammograms (CV) at scan rates ranging from 10 to 70 mV s^−1^ in a non‐faradaic region confirmed the high C_dl_ value of PtO_4_@MX compared to other substrates, following the same tendency in Nyquist plots (Figure S34, Supporting Information).^[^ ^40^ ^]^ However, the discrepancy in mass activity far exceeds that of ECSA, indicating that ECSA is not the predominant factor dictating the overall HER performance. Along with the electrochemical impedance spectroscopy (EIS) results as well as the reduced valence state of Pt, the enhanced catalytic performance of PtO_4_@MX should be attributed to the accelerated electron transfer from MXene to PtO_4_ atomic sites. The resulting electric field from the rapid charge transfer can modulate the Fermi energy, further stabilizing the Pt 5d orbital. Specifically, the resultant high electron density in the Pt 5d orbital reduces the electron density of the O 2p orbitals, thereby weakening the binding energy between oxygen and protons. This facilitates more efficient proton transfer from O to the Pt active sites. Overall, the decreased proton desorption energy not only significantly enhances the overall Tafel process but also ensures a more efficient and sustained supply of protons to the catalytic sites, further optimizing the energy barrier (Figure 3d).

PtO_4_@MX presented a good long‐term stability, attributed to the strong atomic‐level adhesion feature of dopamine. In a chronoamperometry test conducted at −40 mV, the catalyst sustained a stable current density of 20 mA cm^−2^ for over 80 h without any noticeable degradation (Figure 3g). By contrast, the commercial Pt/C catalyst exhibited an inferior stability, with its current density beginning to decline rapidly after 40 h (Figure S35a, Supporting Information). The repeated cycling tests further validated the superior robustness of PtO_4_@MX. After 5000 cycles, the overpotential of PtO_4_@MX increased by only 3 mV (Figure 3h), whereas the commercial Pt/C catalyst exhibited a ≈12 mV increase, indicating a substantial degradation (Figure S35b, Supporting Information). HADDF‐STEM images of PtO_4_@MX acquired after 5000 LSV cycle tests demonstrated that the Pt single atom configuration remained uniform and well‐dispersed, with no evidence of aggregation (Figure S36, Supporting Information). Further structure characterizations using XANES and FT‐EXAFS verified that the Pt atoms retained their Pt─O coordination even after extensive electrochemical cycling (Figure S37, Supporting Information).

### 2.4 Theoretical Investigations

**Figure 4:**
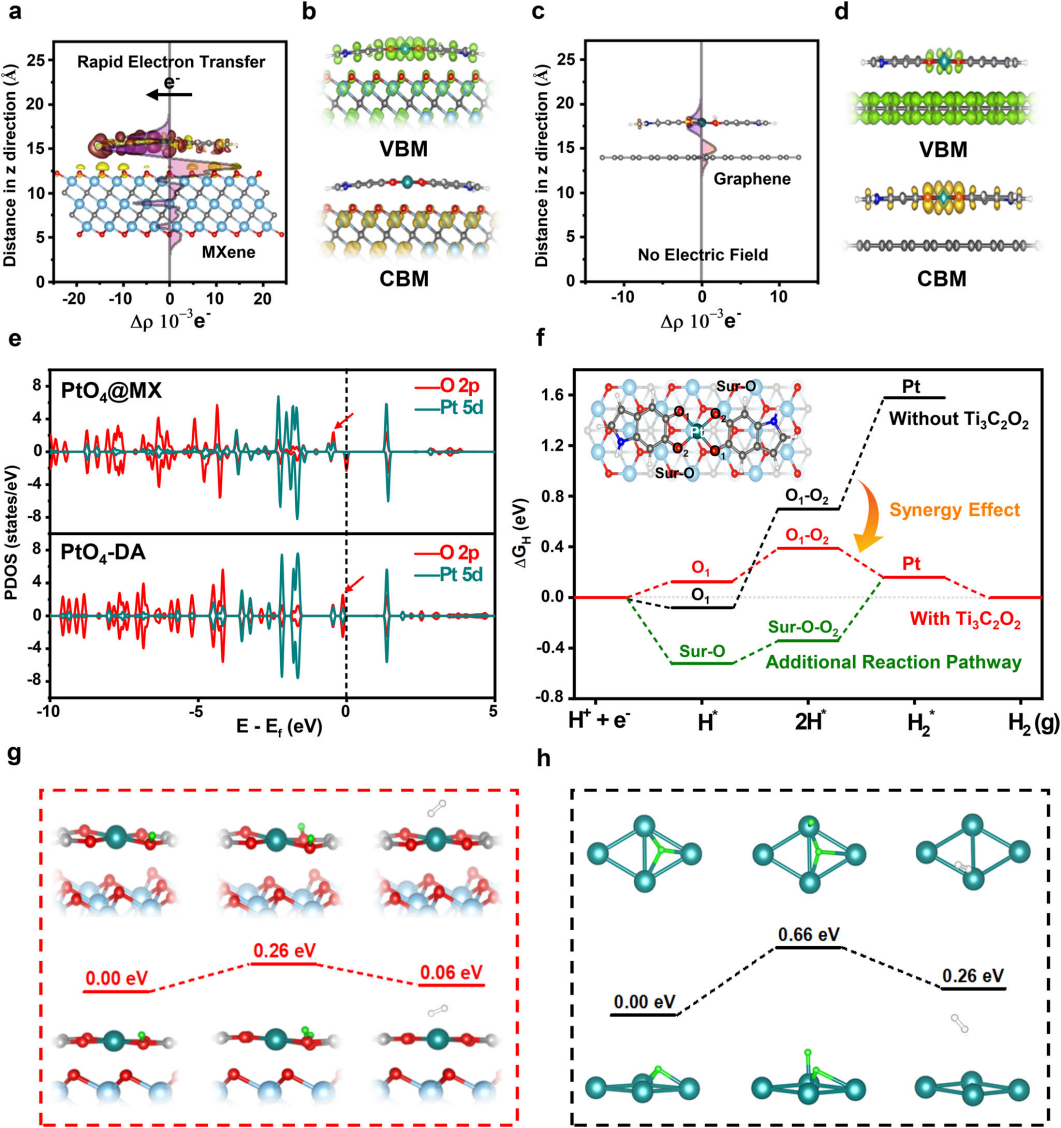
Theoretical investigations. a,c) Charge difference plots with computational models and localized electric field distribution of PtO_4_@MX, and PtO_4_@Graphene. b,d) Electron density distribution at the VBM and CBM of PtO_4_@MX, and PtO_4_@Graphene in real space. e) Calculated PDOS of PtO_4_@MX, and PtO_4_‐DA with aligned Fermi level. f) Free energy diagrams of HER pathways for the Pt─O_4_ active site with and without Ti_3_C_2_O_2_ MXene. g,h) Comparison of the H_2_ evolution kinetics with calculated energy barriers in PtO_4_@MX and Pt (111).

DFT calculations were performed for the atomistic‐level catalytic mechanism of interfacial synergistic HER. Planar PtO_4_ active sites were constructed on Ti_3_C_2_O_2_ MXene, pristine Graphene, and 2H‐MoS_2_ in order to optimize the atomic structure (See Experimental Section for computational details). As a primary factor, the electron transfer is highly dependent on the electron density at the interface. The large Fermi level difference between Ti_3_C_2_O_2_ and PtO_4_‐DA (Table S5, Supporting Information), along with the exceptionally high electron density at the Fermi level of Ti_3_C_2_O_2_, strongly facilitates the electron transfer to the hetero‐interface.^[^ ^41^, ^42^ ^]^ The Bader charge analysis clearly reveals electron accumulation at the hetero‐interface of PtO_4_@Ti_3_C_3_O_2_ (Table S6, Supporting Information). This electron accumulation induces a strong localized electric field at the hetero‐interface, which in turn effectively redistributes the electron density of PtO_4_ (**Figure** **4**a).^[^ ^43^ ^]^ While graphene also possesses a high electrical conductivity, its origin lies in the high charge carrier mobility via delocalized 2p orbitals. Graphene cannot effectively drive rapid electron transfer to the hetero‐interface due to its low charge carrier density, resulting in no significant electric field effect (Figure 4c). The interfacial electric field in PtO_4_@MX increases the electron density of Pt (as demonstrated in the experimental characterizations and DFT calculations), while decreasing the electron density in O, making both more favorable for the Volmer–Tafel process in acidic HER. Although O is generally recognized as an excellent proton‐absorbing site, its binding energy is often excessively strong, impeding the proton desorption. However, the reduced electron density in the O 2p orbital effectively weakens the proton binding energy, allowing an easier proton transfer to the Pt active sites, and thereby significantly reduces the energy required for the Tafel step.

Figure 4b provides the visualization of the electron density corresponding to the valence band maximum (VBM) and conduction band minimum (CBM) of PtO_4_@MX. In our self‐assembled model system, the Fermi levels for the substrates and active sites are effectively separated. The presence of VBM states mainly on the PtO_4_‐DA enables the upper active sites to be optimized for proton adsorption and a subsequent reduction by donating the electrons from the VBM, while the presence of CBM on the underlying substrate is tailored for an efficient electron transfer from the electrode to the catalyst. The intrinsic counterbalancing tendency is well recognized between the Fermi energy required for rapid electron transport via delocalized orbitals and the optimal hydrogen binding energy in localized orbitals.^[^ ^44^ ^]^ Nonetheless, the well‐defined functional separation of the two key features in our PtO_4_@MX enables the unprecedented realization of high electrical conductivity and optimal proton adsorption sites simultaneously. When using graphene as a substrate, its CBM hinders the efficient electron transfer, while the VBM of PtO_4_ active sites remains suboptimal for accelerating HER kinetics (Figure 4d). A similar result is reproduced in the DFT simulation of PtO_4_@MoS_2_, emphasizing the unique optimal feature of PtO_4_@MX for an interfacial synergistic system (Figure S38, Supporting Information).

As illustrated in Figure 4e, the detailed changes in O 2p and Pt 5d orbitals were analyzed through the calculated projected density of state (PDOS) of PtO_4_@MX and PtO_4_‐DA, with their Fermi energy levels aligned. The detailed results exhibit the left shift of PDOS for both O 2p orbital and Pt 5d orbital, indicating the weaker binding strength of the proton intermediate (H^*^) at both atomic sites. The effect of electron redistribution is even more distinct in the free energy diagram (Figure 4f), which clearly displays the detailed HER pathway in PtO_4_ active sites. Without the Ti_3_C_2_O_2_ MXene substrate, the calculated energies for 2H^*^ adsorption and generation of hydrogen intermediate (H_2_^*^) are significantly high, attributed to the strong binding energy of O─H^*^. Thus, the markedly reduced free energy due to the facile electron redistribution clearly supports the synergistic effect of hetero‐interfacial interaction between the Ti_3_C_2_O_2_ substrate and PtO_4_ active sites. Interestingly, the hetero‐interfacial interaction also influences the electrocatalytic property of the MXene substrate, while allowing an additional reaction pathway (Sur‐O‐O_2_, where Sur‐O refers to the Ti_3_C_2_O_2_ surface as defined in Figure 4f) for HER. This unique characteristic mainly arises from the diminished proton adsorption energy at the Sur‐O (from −0.52 to −0.14 eV) and the reduced proton coverage on the Ti_3_C_2_O_2_ surface, induced by the pre‐adsorption of PtO_4_‐DA, which further optimizes the proton binding energy (Figure S39, Supporting Information). It can facilitate proton transfer from MXene to the PtO_4_ active sites and establish abundant pathways for H^*^ supply. As a result of these synergistic effects, PtO_4_@MX achieves a remarkably low energy barrier of 0.26 eV in the rate‐determining step of HER, as calculated in Figure 4g. This offers superior HER kinetics compared to Pt (111) with a higher energy barrier of 0.66 eV for the same rate‐determining step (Figure 4h). In conjunction with the experimental results, DFT simulation successfully confirms the fluent electron transfer, additional reaction pathway, and optimized energy barrier originating from the ideal interfacial HER, as illustrated in Figure 3d.

## 3 Conclusion

We have demonstrated an innovative molecular self‐assembly approach from dopamine precursors as an adhesive module to construct structurally precise and stable atomic catalytic sites. This intriguing mechanism facilitates the controlled and uniform monolayer deposition of monodisperse PtO_4_ atomic sites across various substrate surfaces. Taking advantage of these unique features, we systematically investigated the beneficial mechanisms of hetero‐interfacial interactions in enhancing the HER kinetics, as solely driven by the physical nature of substrate materials. This mechanistic study is uniquely feasible in our self‐assembled system without any specific binding between the substrate and the active site, yet well‐securing their intimate interplay. Notably, a remarkable enhancement was observed in the mass activity of PtO_4_ sites, up to 30‐fold increase from the commercial Pt/C reference. Specifically, the hetero‐interface between the electron‐rich MXene and the PtO_4_ atomic sites readily facilitates the efficient electron transfer and builds up a strong localized electric field. This further increases the electron density in Pt 5d orbital, while decreasing the hydrogen binding energy in the O 2p orbital, and thereby optimizes the hydrogen adsorption/desorption at the PtO_4_ sites. These findings highlight the critical role of noncovalent environmental interaction upon electrocatalytic activity, which has been difficult to systematically explore thus far, and also open‐up a valuable pathway toward superior catalytic performance by judiciously pairing atomic catalytic sites with the substrates of distinct synergistic features.

## 4 Experimental Section

### Synthesis of Delaminated Ti_3_C_2_T_x_ MXene

Ti_3_AlC_2_ MAX powder (particle size: 40 µm) was acquired from Carbon‐Ukraine Ltd (Ukraine). Hydrofluoric acid (HF, 48%) was purchased from Alfa Aesar (USA). Hydrochloric acid (HCl, 37%) and Lithium chloride (LiCl, 99%) were supplied by Sigma–Aldrich (USA). Briefly, 3.0 g of MAX powder was added to a 60 mL of 6:3:1(volume ratio) mixture of 12 m HCl, deionized (DI) water, and 48 wt.% HF for the etching process. After fully eliminating aluminium layers by stirring with etchant at 400 rpm for 24 h at 36 °C, the etched multilayer MXene was washed 4–5 times with DI water to remove the HF and remaining metal ions. The resulting sediment was then dispersed in 150 mL of 0.5 m LiCl solution and stirred at 400 rpm for 4 h at room‐temperature. After the delamination process, the MXene/LiCl mixture was repeatedly centrifuged with DI water (6000–10,000 rpm) until the pH of the supernatant became 5–6. In the final washing cycle, 1 or 2‐layer MXene solution can be collected as a supernatant after 20 min of centrifugation at 4000 rpm.

### Preparation of NrGO

NRGO was supplied by Graphene All (Republic of Korea). In order to maximize electrical conductivity while preserving excellent dispersibility, Graphene Oxide (GO) was reduced via ammonia solution reduction rather than conventional thermal methods or plasma reduction. Furthermore, to selectively obtain highly dispersible flakes, the NrGO dispersion was left undisturbed for 1 h, allowing the sedimented particles to settle, after which only the supernatant was carefully collected for further experiments.

### Synthesis of Delaminated MoS_2_

Bulk MoS_2_ and n‐Butyllithium were purchased from Sigma–Aldrich (USA). 0.3 g of bulk MoS_2_ was added to 6 mL of 2.5 m n‐Butyllithium in hexane solution, and stirred at 300 rpm for 48 h at 40 °C. The fully intercalated MoS_2_ suspension was filtered over a Millipore membrane and repeatedly washed with high‐purity hexane to remove the residual n‐Butyllithium. The aforementioned procedures were carried out in an N_2_‐filled glove box. Then, the sediments were centrifuged several times with DI water at 17 000 rpm in order to fully remove the hexanes. In the final washing cycle, 1 or 2‐layer MoS_2_ aqueous solution can be collected as a supernatant after 30 min of centrifugation at 3000 rpm.

### Synthesis of PtO_4_‐DA

Chloroplatinic acid (H_2_PtCl_6_) and dopamine hydrochloride (DA) were obtained from Sigma–Aldrich (USA). To prepare PtO_4_‐DA, 89.2 mg of H_2_PtCl_6_ and 200 mg of DA were added to 50 mL deoxygenated DI water solution, and stirred at 400 rpm for 4 h in acidic conditions. It is important to note that the commonly used Tris buffer solution should not be used in order to minimize the possibility of rapid dopamine polymerization during the chelation reaction. Then, 1 mL of 1 wt.% NH_4_OH was added dropwise to slowly initiate the self‐oxidation of dopamine, simultaneously facilitating the reduction of chelated platinum ions. All the procedures were conducted in room‐temperature with ambient solution conditions. After 1 h stirring and 10 min sonication, the resulting solution was washed several times at 17 000 rpm with ethanol and DI water to separate the fabricated PtO_4_‐DA from excessive dopamine monomers and ion impurities. In the final washing cycle, atomically dispersed PtO_4_‐DA can be collected as a supernatant after 10 min of centrifugation at 3000 rpm.

### Synthesis of PtO_4_@MX, PtO_4_@NrGO and PtO_4_@MoS_2_

To prepare PtO_4_@MX, a predetermined amount of PtO_4_‐DA was added to the MXene aqueous solution with the concentration of 0.1 mg mL^−1^. The pH condition of MXene was controlled to 3.5 by using 0.5 m HCl to induce the opposite zeta potential between MXene and PtO_4_‐DA. Since the mixture was not a buffered solution, its pH could fluctuate within the range of 3.0–3.5 over time. The minus‐charged 2D MXene flakes could be readily self‐assembled with plus‐charged PtO_4_‐DA during the 2 h of stirring at 400 rpm. After three times of repeated centrifugation and decanting, PtO_4_@MX was obtained as a resulting sediment. The equal procedures and conditions were required for the fabrication of PtO_4_@NrGO and PtO_4_@MoS_2_ as a predetermined amount of different 2D nanomaterials could electrostatically self‐assemble with PtO_4_‐DA. Facile pH control was essential for the uniform dispersion of PtO_4_‐DA on substrates. All samples were freeze‐dried to obtain the powder and stored in a vacuum desiccator to minimize oxidation.

### Synthesis of Pt@MX

A predetermined amount of H_2_PtCl_6_ was added to the MXene aqueous solution, stirred at 400 rpm for 4 h. Then, an excessive amount of 1 mg mL^−1^ NaBH_4_ (strong reducing agent) solution was added dropwise to fully reduce the platinum precursors. After repeated centrifugation at 10 000 rpm and decanting, Pt@MX was obtained.

### Synthesis of PtO_4_‐PDA_NP_ and PDA_NP_

89.2 mg of H_2_PtCl_6_ and 200 mg of DA were added to 50 mL deoxygenated DI water solution, and stirred at 400 rpm for 4 h in acidic conditions. Then, 50 mL of 0.02 m Tris buffer solution was added, and the pH was adjusted to 8.5 using a 0.5 m NaOH solution. The mixture was stirred at 400 rpm for 24 h under ambient conditions, allowing the formation of PDA nanoparticles. After repeated centrifugation at 10 000 rpm and decantation, PtO_4_‐PDA_NP_ was obtained as the sediment. PDA_NP_ was synthesized following the same procedure, with the exception that H_2_PtCl_6_ precursors were not used.

### Characterizations

Zeta potential measurements were conducted in aqueous solution with the same concentration of 0.01 mg mL^−1^ using ELSZ‐2000 (Otsukael). All the pH values were measured accurately using AB33PH (OHAUS) after calibration. The XRD spectra were obtained using a SmartLab (RIGAKU) diffractometer with a scanning step of 0.01° and a scan rate of 5° min^−1^. High‐resolution Raman was performed using LabRAM HR Evolution Vis–NIR (HORIBA) with a wavelength laser of 514 nm. XPS spectra were acquired using K‐alpha equipment (Thermo VG Scientific) with an aluminum X‐ray source (1486.7 eV). All the binding energy values were compared after applying carbon correction based on the C 1s spectrum to compensate for peak shifts caused by charging effects. FT‐IR was conducted using Nicolet iS50 (Thermo Fisher) without pellet preparation. The thickness of 2D nanomaterials was measured by INNOVA‐LABRAM HR800 (Horiba Jobin Yvon), and the Pt content of the catalysts was measured using ICP‐OES 720 (Agilent). The morphological characterizations and EDS analysis were investigated by Spectra Ultra (Thermo Fisher), which was a High‐Resolution Double Cs corrected TEM, at 300 kV accelerating voltage. BET isotherms were obtained through N_2_ adsorption–desorption measurements using a 3Flex (Micromeritics). The BET‐specific surface area was calculated based on adsorption data within a selected relative pressure range of P/P₀ = 0.05–0.3. In order to verify the atomic structures of the PtO_4_ catalytic sites, X‐ray absorption fine structure (XAFS) measurements at Pt L_3_‐edge were performed using the 8C beamline of Pohang Accelerator Laboratory (PLS‐II, 3.0GeV0, South Korea). Pt foil was used as a reference sample for the precise energy calibration. The obtained XANES and EXAFS data were analysed using ATHENA software, following standard analytical procedures. The fitting results were obtained from the k^3^‐weighted EXAFS oscillation using the ARTEMIS module, incorporating Feff calculations for the optimized PtO_4_ structure.

### Electrochemical Measurements

All electrochemical analyses were carried out on an electrochemical workstation (VSP, Bio‐logic) at room‐temperature using a three‐electrode system with a Pt wire as the counter electrode, a 3.0 m Ag/AgCl electrode as the reference electrode, and carbon paper as the working electrode. 0.5 m H_2_SO_4_ was used as an electrolyte, and the reference electrode was calibrated to reversible hydrogen electrode (RHE), *E*_RHE_  =  *E*_Ag/AgCl_ + 0.059  ×  pH + 0.210 V. The electrode ink was prepared by mixing 5 mg of catalyst with 1 mL of solvent (1:1 v/v water/ethanol) and 10 µL of Nafion (10 wt.%), followed by grinding and sonication. Subsequently, 200 µL of the prepared ink was precisely deposited onto the 1 cm^2^ carbon paper (Thermo Scientific) and subjected to vacuum drying. All the catalytic inks, including the 40 wt.% commercial Pt/C, were fabricated using the identical preparation procedures and drop‐cast in equal amounts to ensure consistency. The linear sweep voltammetry (LSV) curves were measured at a scan rate of 5 mV s^−1^, with all data calibrated by 95% iR‐compensation within the electrochemical cell. The EIS spectra were performed at an overpotential of 10 mV, in the frequency range from 100 kHz to 10 mHz. Any possibility of Pt contamination was eliminated by conducting identical experiments using a carbon rod counter electrode as a control.

### Tafel Slope, Mass Activity, and Double Layer Capacitance Calculations

The Tafel slopes were determined by performing linear fitting on the plot of logarithmic current density versus overpotential. Tafel equation:

(1) $$\eta =b\log\left(j\right)+c$$

where b is the Tafel slope, η is the overpotential (mV), j is the measured current density (mA cm^−2^), and c is the intercept. The mass activity of the catalysts was calculated using the following equation:

(2) $$\mathrm{mass}\;\mathrm{activity}\;=j/m_{\mathrm{Pt}}$$

where j is the measured current density (mA cm^−2^), and m_Pt_ is the platinum loading (mg cm^−2^). Cyclic voltammograms (CV) were performed in the potential range of 0.2–0.3 V (vs RHE) with the scan rates from 10 to 70. mV s^−1^ to determine the double layer capacitance (C_dl_) of the catalysts. C_dl_ was calculated using the following equation:

(3) $$C_{\mathrm{dl}}=\frac{\Delta j\;/2}{\Delta \nu }$$

where Δj is the difference of the anodic and cathodic currents (j_a_–j_c_) at 0.25 V versus RHE and Δν is the scan rate difference.

### Computational Details

A self‐consistent approach within the density functional theory (DFT) framework, as implemented in the Vienna Ab initio Simulation Package (VASP),^[^ ^45^ ^]^ was employed to model and optimize a monolayer of Ti_3_C_2_O_2_ along with a PtO_4_‐DA adsorbed Ti_3_C_2_O_2_ monolayer. The electronic wave functions were described using the projector‐augmented wave (PAW) pseudo‐potential formalism,^[^ ^46^ ^]^ while generalized gradient approximations (GGA) were utilized to approximate the electron‐electron correlation functionals.^[^ ^47^, ^48^ ^]^ The electronic optimizations were performed with a convergence criterion of 10^−6^ eV, and ionic relaxations were iteratively executed until the absolute forces between all atoms were reduced to less than 0.01 eV Å^−1^. The monolayer model of Ti_3_C_2_O_2_ was constructed to be periodic along the x‐ and y‐directions, with a 20 Å vacuum in the z‐direction to prevent interactions between periodic images. The cell parameters of the monolayer models were not optimized, but atomic positions were fully relaxed based on the aforementioned criteria. For structural relaxation, the Brillouin zone was sampled with a 16 × 16 × 1 Γ‐centered k‐point mesh, while a 3 × 3 × 1 k‐point grid was used for the 5 × 5 × 1 supercell. The density of states (DOS) calculations were performed via non‐self‐consistent calculations, utilizing charge density and wavefunctions obtained from self‐consistent calculations.

Gibb's free energy calculation: To evaluate the HER catalytic activity of various active sites on the PtO_4_‐DA adsorbed Ti_3_C_2_O_2_ monolayer, the computational hydrogen electrode (CHE) model, developed by Nørskov et al.^[^ ^49^ ^]^was employed. The Gibbs free energy change (ΔG) for hydrogen adsorption was calculated using the expression:

(4) $$\Delta G_{H^{*}}=\Delta E_{H^{*}}+\Delta E_{\mathrm{ZPE}}-T\Delta S_{H}$$

where Δ*E*, *T*, Δ*S*, and ZPE denote the change in enthalpy upon hydrogen adsorption (computed from the DFT total energy of the system with and without an adsorbed H atom), the absolute temperature, the entropy changes of the adsorbed hydrogen, and the zero‐point energy correction, respectively. Standard entropy values for molecular H_2_ were sourced from the NIST database, while those for adsorbed hydrogen atoms were derived from their vibrational frequencies.

Binding Energy Calculation: To compare the binding energies of covalent bonding and Pt─Pt metallic bonding, the Pt─Pt bond strength was systematically varied by adjusting the Pt concentration, ranging from a Pt‐poor condition, where a low Pt concentration leads to the formation of Pt_2_ dimers, to a Pt‐rich condition, where a high Pt concentration enables the formation of bulk Pt particles. The binding energy (BE) is determined using the following equation:

(5) $$\mathrm{BE}\;=E\left(\mathrm{Str}+\mathrm{Pt}\right)-E\left(\mathrm{Str}\right)-\mu \left(\mathrm{Pt}\right)$$

where *E* (Str+Pt) represents the total energy of the structure with a Pt atom, *E* (Str) denotes the total energy of the pristine structure (without Pt), and *µ* (Pt) corresponds to the chemical potential of a Pt atom. Here, the chemical potential of Pt in the Pt‐poor condition (Pt dimer state) was set to 0 eV.

## Conflict of Interest

The authors declare no conflict of interest.

## Author Contributions

Y.H.Y. led the project and mainly carried out the experiments. K.J. carried out the DFT calculations and commented on the mechanistic understanding. G.S.L. contributed to the structure characterizations and commented on the experiments. J.B. and S.P.S. contributed to the electrochemical measurements. C.W.L., C.C., and J.B.K. contributed to the material synthesis. H.L. commented to the interpretation of the dopamine mechanism. S.O.K. supervised and wrote the paper.

## Supporting information



Supporting Information

## Data Availability

The data that support the findings of this study are available on request from the corresponding author. The data are not publicly available due to privacy or ethical restrictions.
