# Supplementary material for: Mussel‐Inspired Self‐Assembly of PtO4 Atomic Catalysts for Interfacial Synergistic Hydrogen Evolution
Source: Adv Sci (Weinh). 2025 Jun 29;12(37):e07807. doi: 10.1002/advs.202507807 (PMC12499478; doi:10.1002/advs.202507807)
Supplement: Supplementary file 1 — Supporting Information [file ADVS-12-e07807-s001.docx]

**Supporting Information**

**Mussel-Inspired Self-Assembly of PtO_4_ Atomic Catalysts for Interfacial Synergistic Hydrogen Evolution**

*Yeo Hoon Yoon, Karthikeyan Jeyakumar, Gang San Lee, Jayaraman Balamurugan, Suchithra Padmajan Sasikala, Chan Woo Lee, Colin Wing-Lok Cheng, Jun Beom Kim, Haeshin Lee and Sang Ouk Kim**

Mr. Y. H. Yoon, Dr. G. S. Lee, Prof. J. Balamurugan, Prof. S. P. Sasikala, Mr. C. W. Lee, Mr. C. Cheng, Mr. J. B. Kim, and Prof. S. O. Kim

National Creative Research Initiative Center for Multi-Dimensional Directed Nanoscale Assembly, Department of Materials Science and Engineering, KAIST, Daejeon 34141, Republic of Korea.

Prof. K. Jeyakumar

Department of Physics, National Institute of Technology, Durgapur, West Bengal 713209, India.

Prof. H. Lee

Department of Chemistry, Korea Advanced Institute of Science and Technology, Daejeon 34141, Republic of Korea.

*E-mail of corresponding author: sangouk.kim@kaist.ac.kr


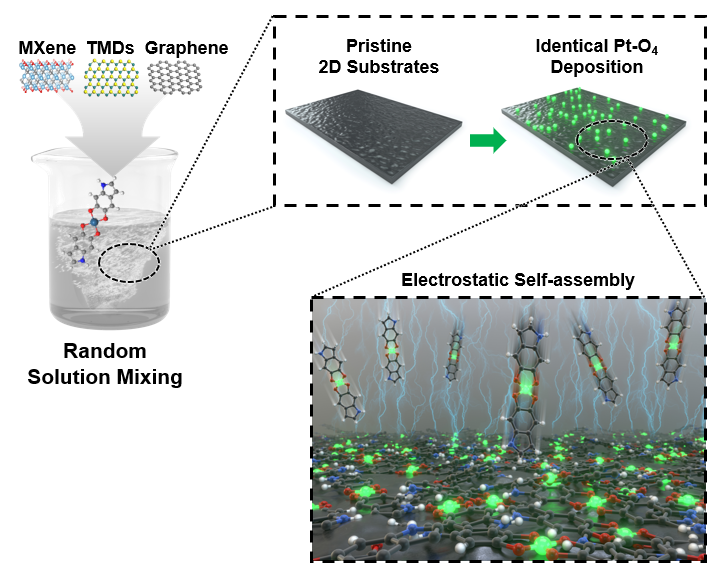


**Figure S1**. Schematic illustration of the deposition of identical PtO_4_ atomic catalytic sites onto various 2D substrates via random solution-phase mixing.

By employing dopamine as a molecular module, PtO_4_ atomic catalytic site was functionalized with its intrinsic adhesive properties through a self-assembly process, imparting strong affinity toward a broad spectrum of water-dispersible materials, including MXene, TMDs, and graphene. Achieving uniform dopamine dispersion across diverse substrates while suppressing undesirable aggregation or multilayer stacking requires precise pH regulation. However, under excessively acidic conditions, ionic charge screening can interfere with electrostatic interactions, thereby disrupting homogeneous dispersion and undermining overall system stability. This mechanism indicates that dopamine-mediated adhesion may be extended beyond 2D nanomaterials to include metal particles and metal oxides, provided that sufficient dispersion stability is maintained in aqueous media.


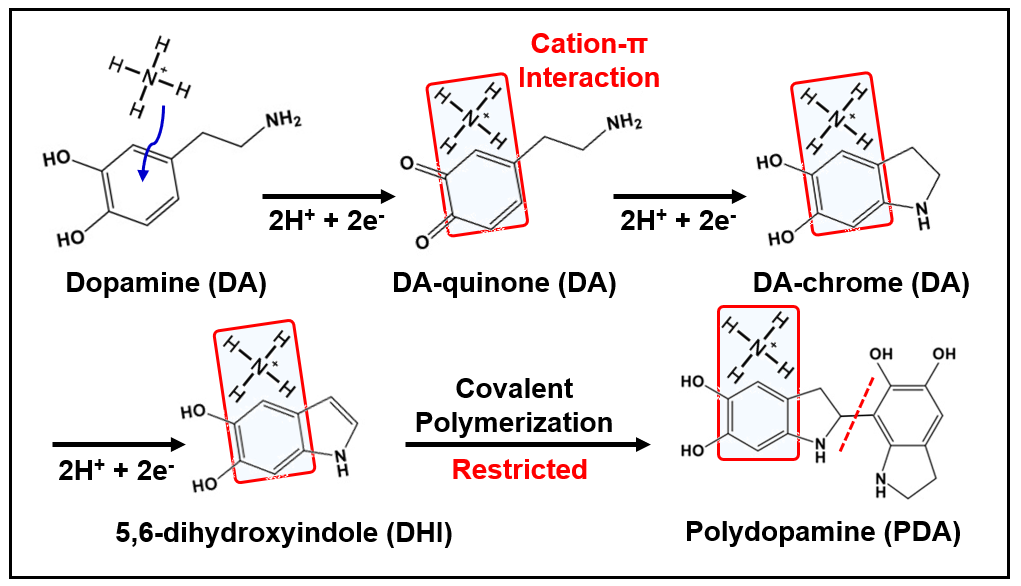


**Figure S2**. Proposed mechanism of dopamine self-oxidation and the suppression of covalent polymerization via cation–π interactions.

The key determinant in the synthesis of PtO_4_-DA is the inhibition of dopamine polymerization. Given that dopamine inherently exhibits low electrical conductivity, it must be directly self-assembled on substrates as an atomic layer to optimize its catalytic performance, thereby enabling its application as an interfacial synergistic HER. In this study, we strategically introduced dropwise NH_4_OH in acidic conditions to promote the initial self-oxidation and cyclization of amine chains while effectively inhibiting the subsequent covalent polymerization (Figure S3). Specifically, adsorbed NH_4_^+^ cations on the dopamine ring localize electrons through cation-π interactions, effectively inhibiting excessive electron transfer required for covalent polymerization. Consequently, the controlled suppression of polymerization favors the dominant formation of DA and DHI throughout the process (Figure S9), facilitating the precise synthesis of PtO_4_-DA and the uniform atomic-layer coating on substrates (Figure 2c).


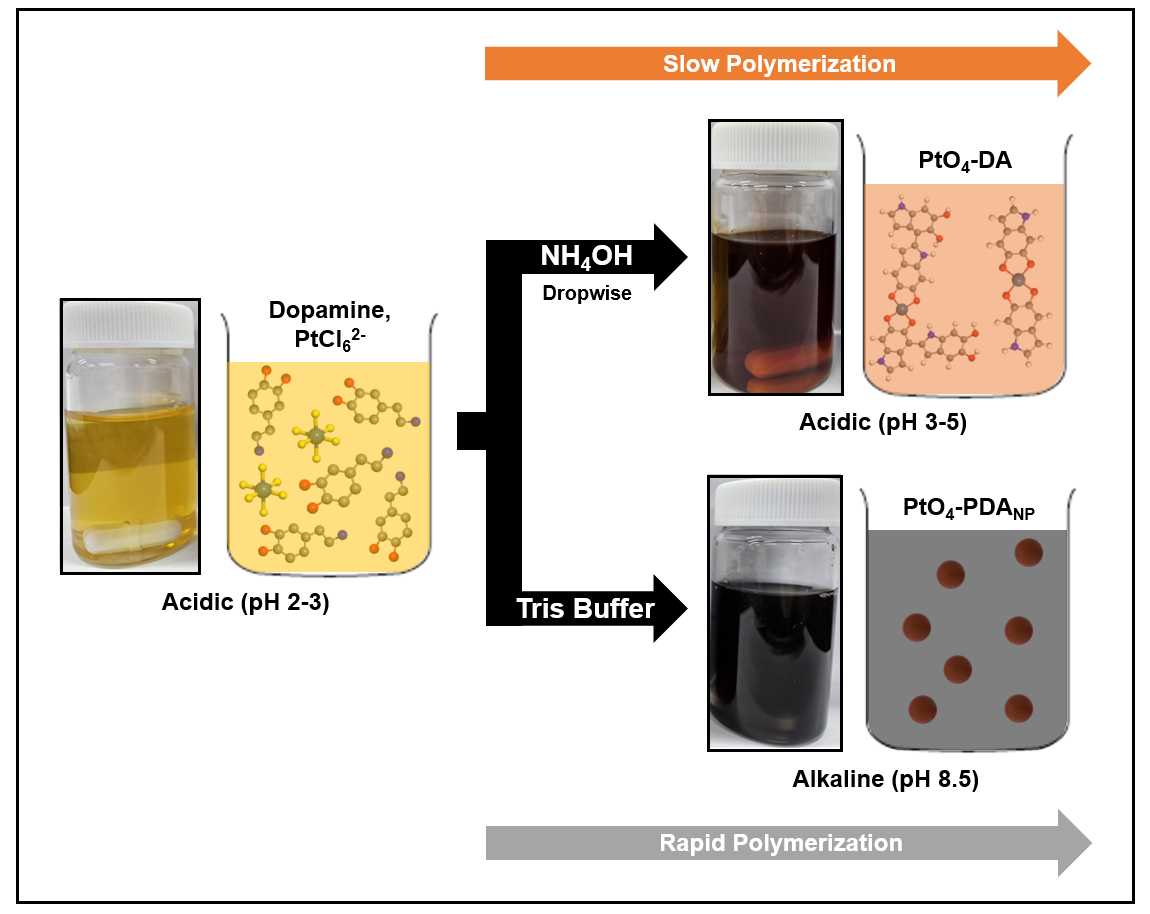


**Figure S3.** Schematic representation of the room temperature molecular self-assembly of PtO_4_-DA compared to the formation of PtO_4_-PDA_NP_ with different procedures.


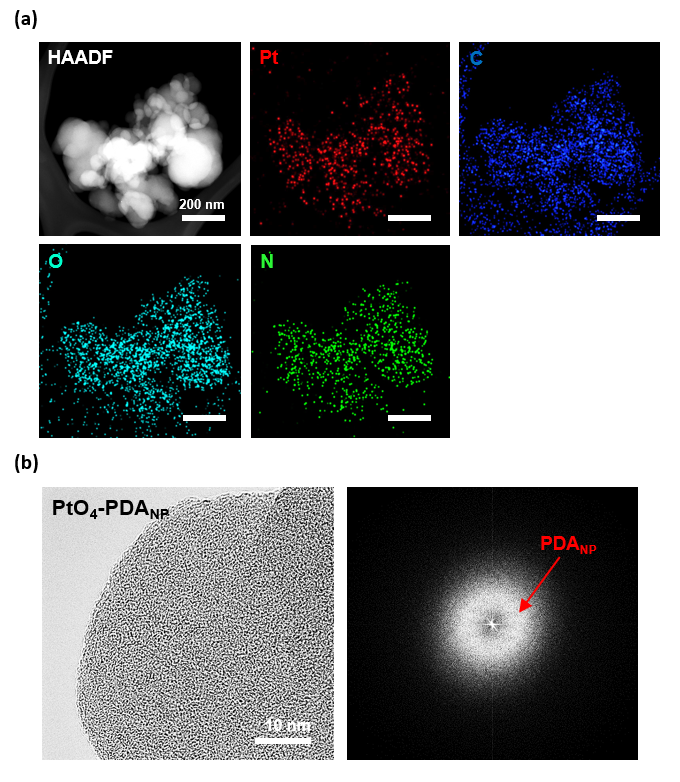


**Figure S4. (a)** HAADF-STEM image of fabricated PtO_4_-PDA_NP_ and the elemental mapping of the corresponding elementals: Pt, C, O, and N. **(b)** HRTEM (High Resolution TEM) image of PtO_4_-PDA_NP_ with the corresponding FFT pattern.

**
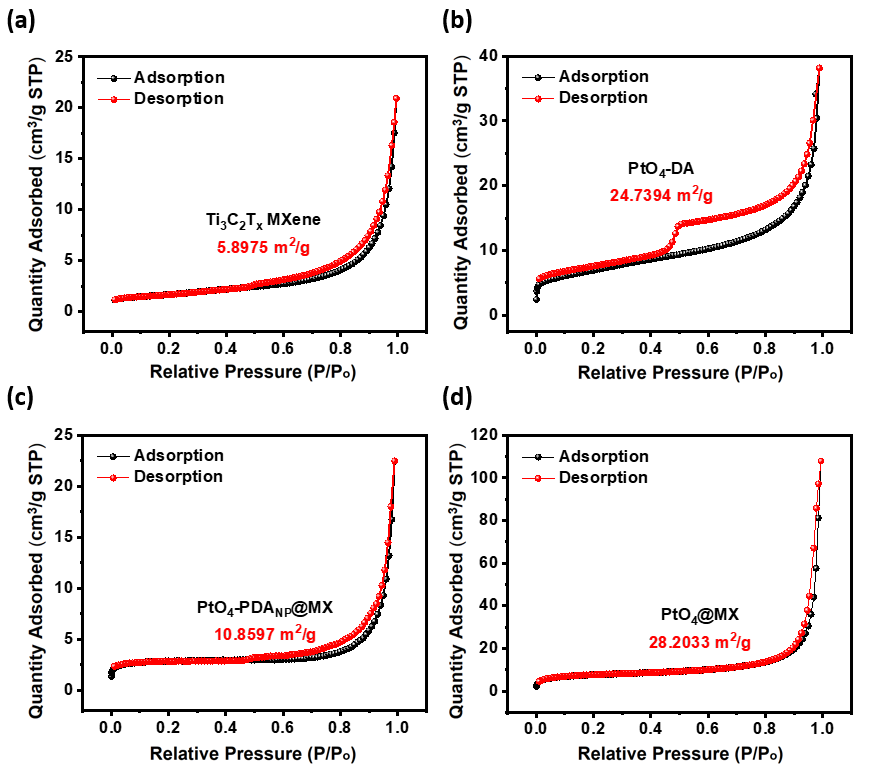
**

**Figure S5**. Nitrogen adsorption/desorption isotherms and BET surfaces areas of **(a)** Ti_3_C_2_T_x_ MXene, **(b)** PtO_4_-DA, **(c)** PtO_4_-PDA_NP_@MX, and **(d)** PtO_4_@MX, respectively.


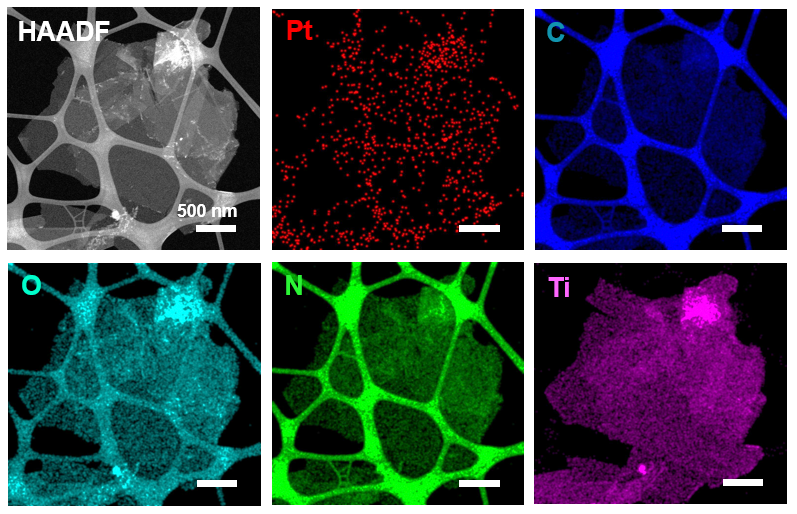


**Figure S6.** HAADF-STEM image of fabricated PtO_4_@MX and the elemental mapping of the corresponding elementals: Pt, C, O, N, and Ti.

The PtO_4_@MX sample was synthesized under optimized conditions, and all characterization techniques, including TEM, XANES, EXAFS, XPS, and XRD, along with HER performance measurements, were conducted on the same PtO_4_@MX sample to ensure consistency in analysis.


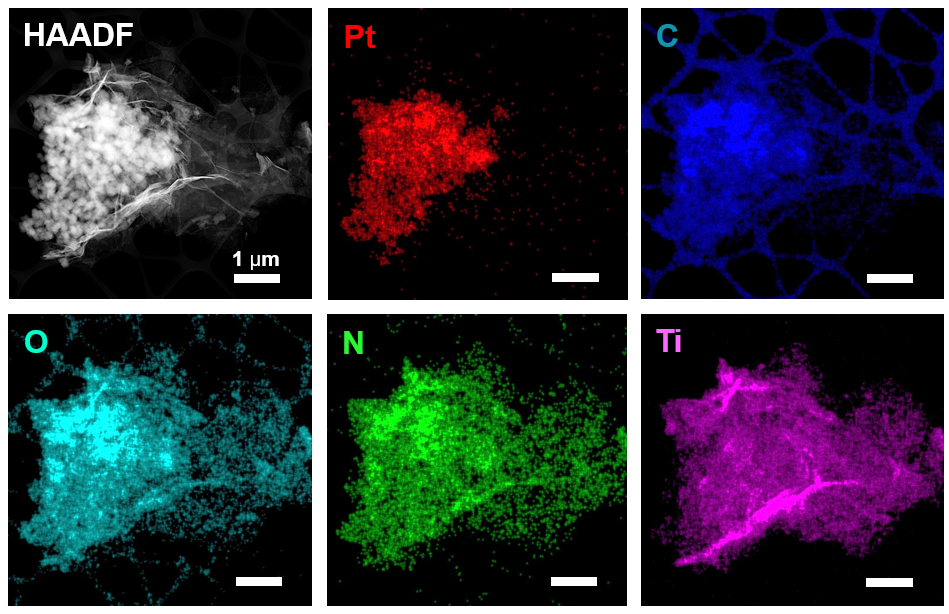


**Figure S7.** HAADF-STEM image of fabricated PtO_4_-PDA_NP_@MX and the elemental mapping of the corresponding elementals: Pt, C, O, N, and Ti.


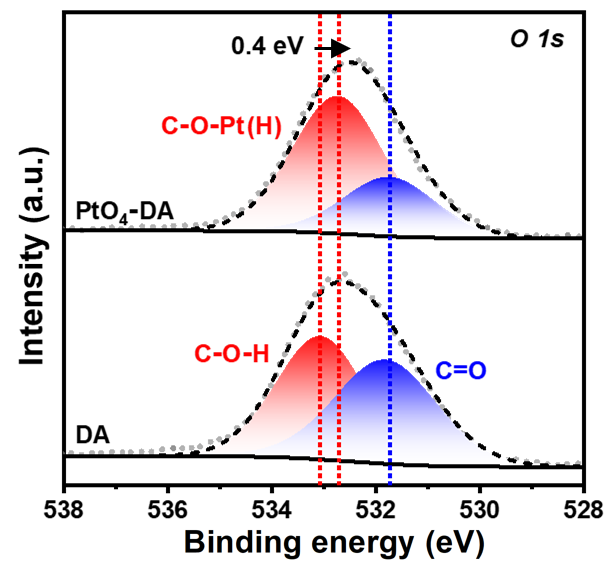


**Figure S8.** XPS O 1s spectra of DA and PtO_4_-DA with displaying deconvolution peaks for C-O and C=O bonds.

To accurately compare the binding energy differences in the XPS O 1s spectra, DA was fabricated without H_2_PtCl_6_, while maintaining the same self-oxidation condition using NH_4_OH. Both datasets were acquired under identical analysis conditions with a uniform sampling procedure. Additionally, carbon correction was performed using the C-C, C=C binding energy (284.6 eV) from the XPS C 1s spectra (Figure S9) to ensure consistency and accuracy.

**
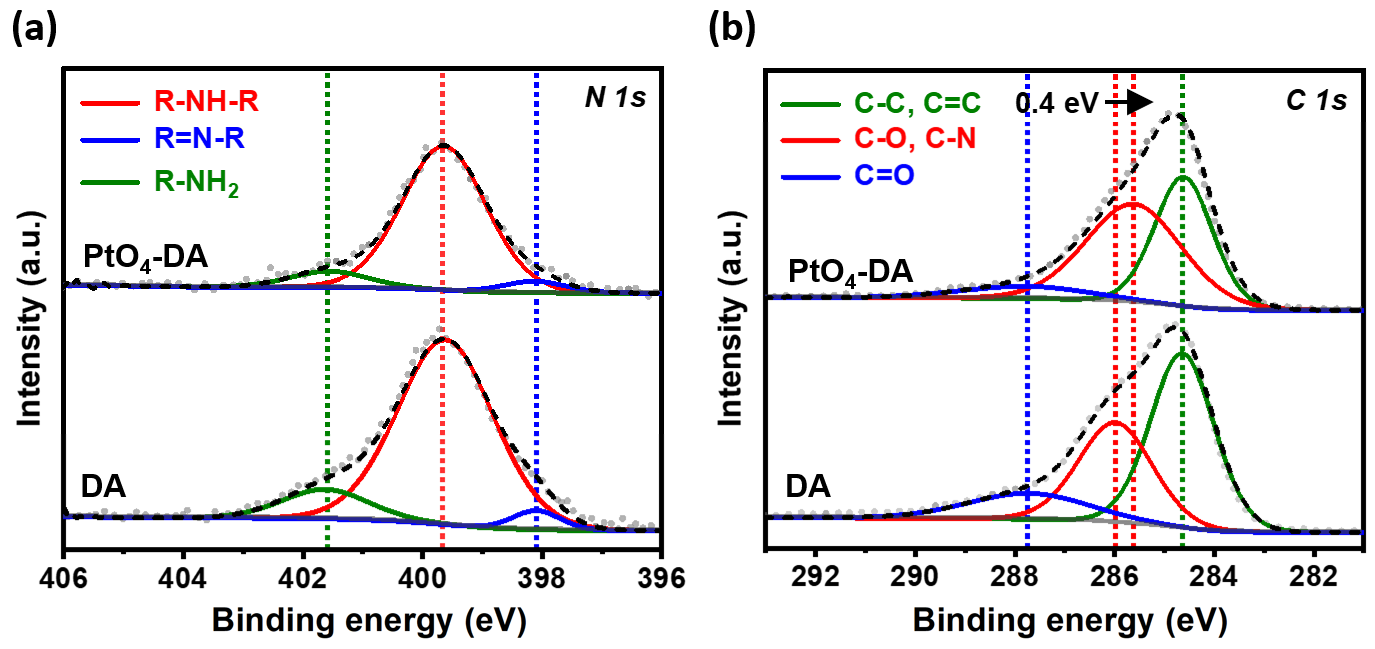
**

**Figure S9.** XPS **(a)** N 1s spectra, and **(b)** C 1s spectra for DA and PtO_4_-DA with displaying deconvolution peaks.

In the XPS N 1s spectra, both DA and PtO_4_-DA exhibit a predominant R-NH-R peak, indicating the oxidation and cyclization of dopamine following the proposed reaction process. The absence of Pt-N peak in PtO_4_-DA indicates the dominant Pt-Catechol group chelation reaction in our proposed procedure. In the XPS C 1s spectra, while the C-C, C=C, and C=O deconvoluted peaks remained unchanged, the C-O peak exhibited a slight shift of 0.4 eV, demonstrating a selective binding energy change induced by the chelation reaction of the catechol groups.

**
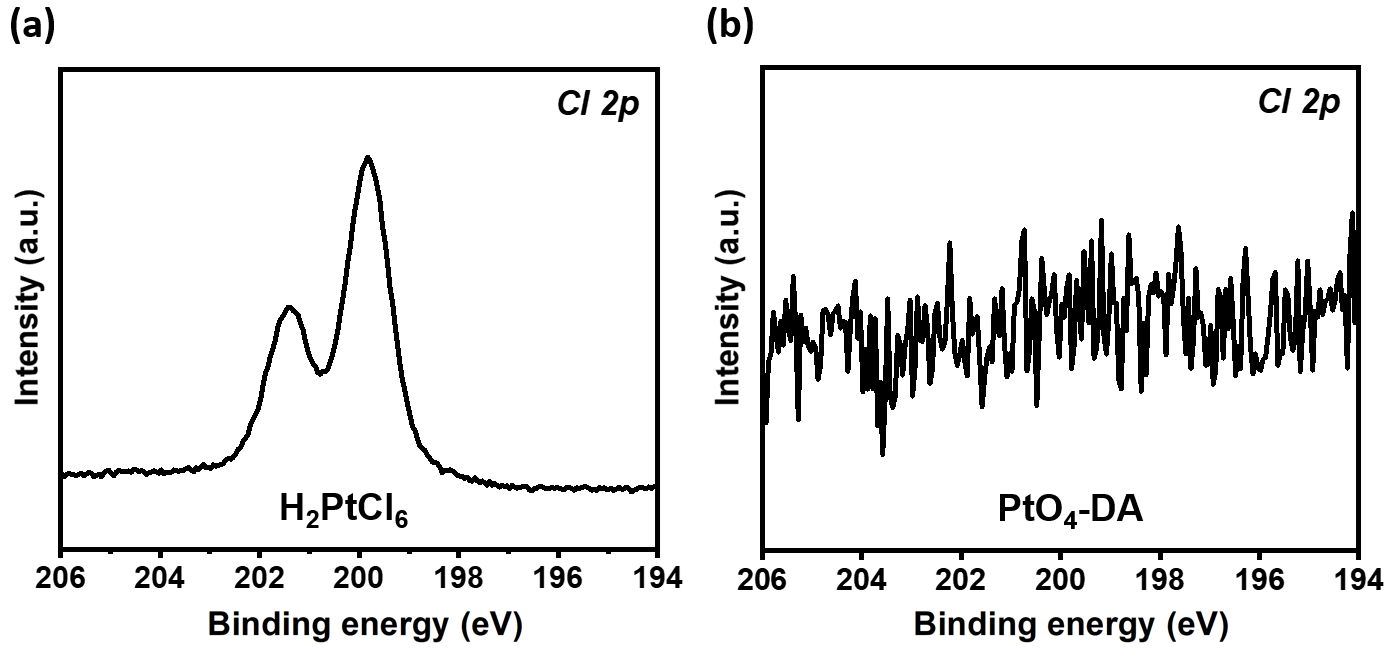
**

**Figure S10.** XPS Cl 2p spectra of **(a)** H_2_PtCl_6_ precursor, and **(b)** PtO_4_-DA.


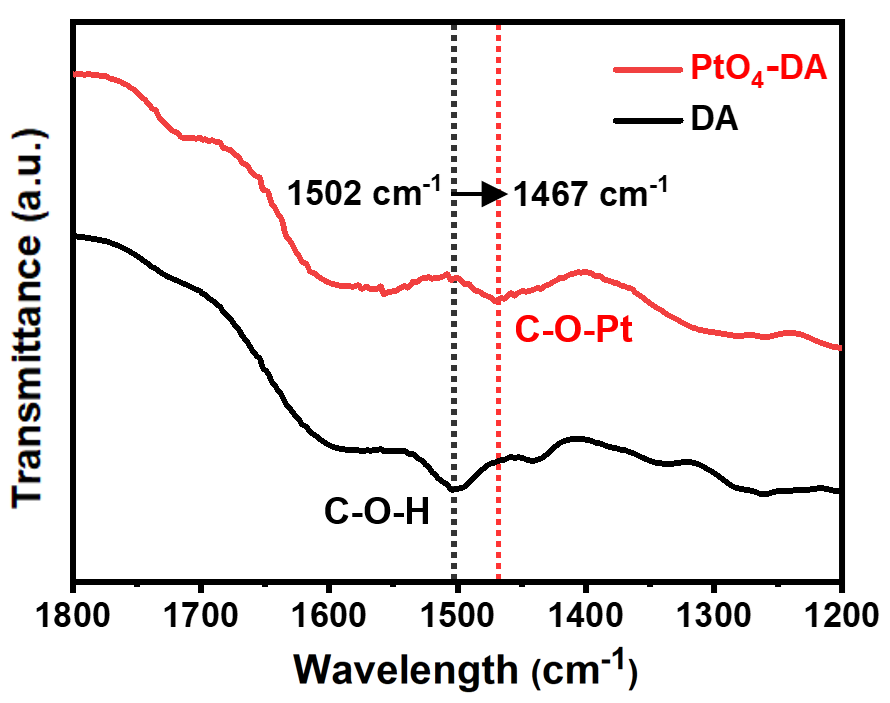


**Figure S11.** FT-IR spectra of PtO_4_-DA and DA.


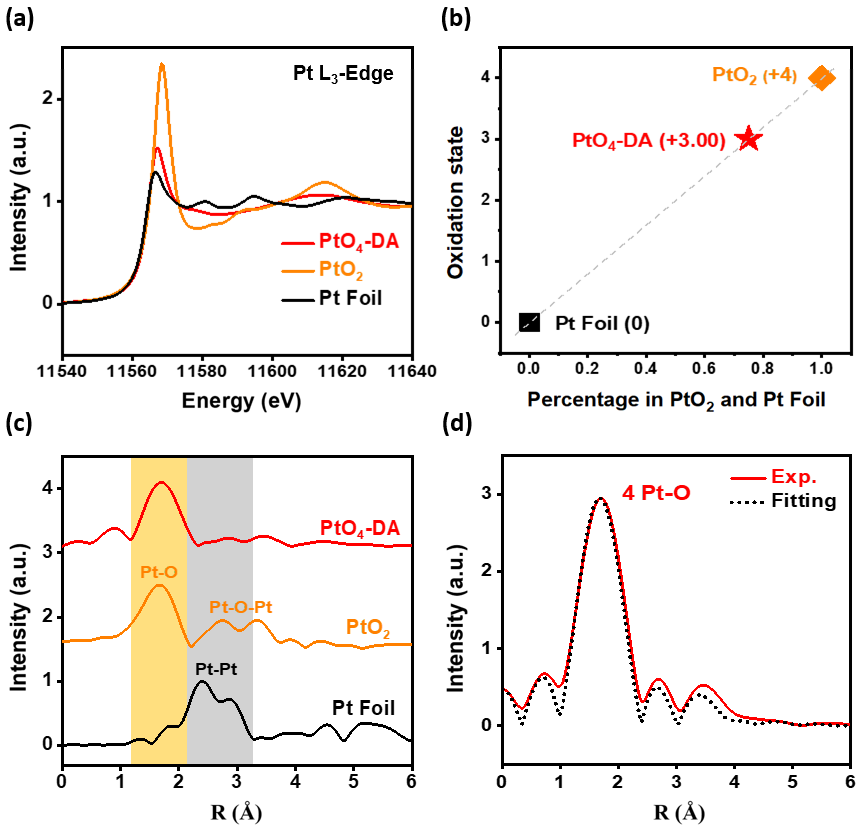


**Figure S12. (a)** XANES spectra at Pt L_3_-edge of PtO_4_-DA, PtO_2_, and reference Pt foil. **(b)** Corresponding FT-EXAFS results. **(c)** Calculated oxidation state of PtO_4_-DA (+3.00) with the relative contribution based on reference Pt foil (0) and PtO_2_ (+4). **(d)** FT-EXAFS fitting curve of PtO_4_-DA with only 4 Pt-O pathways.


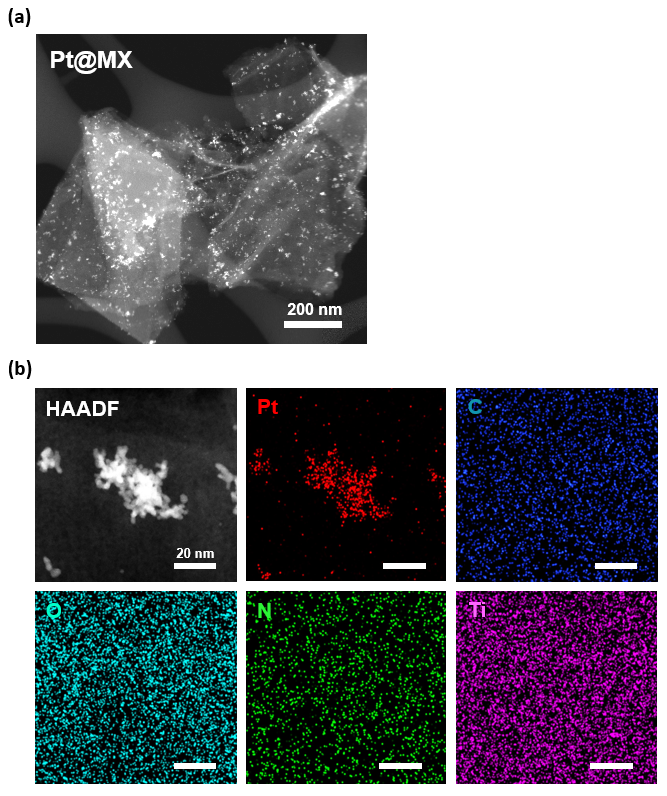


**Figure S13. (a)** HAADF-STEM image of fabricated Pt@MX. **(b)** The magnified HAADF-STEM image and the elemental mapping of the corresponding elementals: Pt, C, O, N, and Ti.


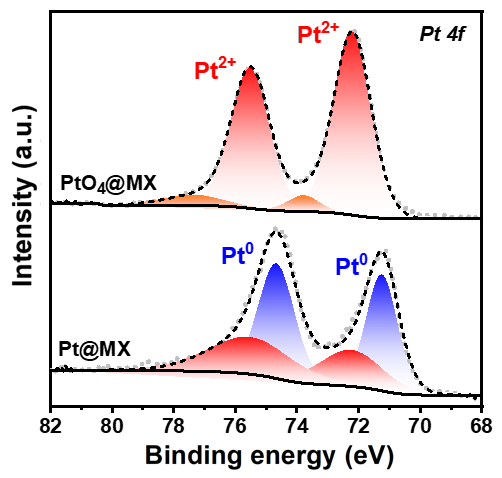


**Figure S14.** XPS Pt 4f spectra of Pt@MX and PtO_4_@MX with deconvolution peaks for Pt^2+^ and Pt^0^.

**
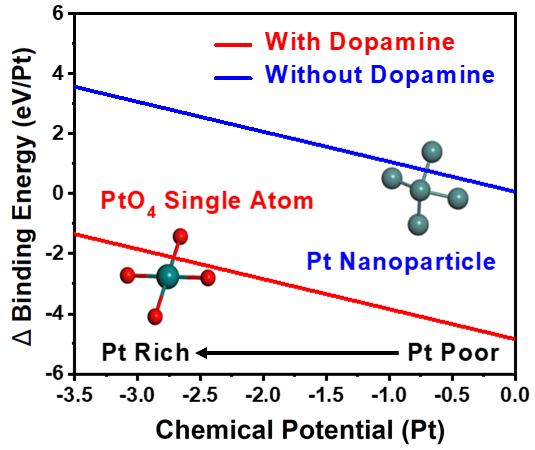
**

**Figure S15.** DFT calculation for the binding energy difference between Pt-O covalent bonding and Pt-Pt metallic bonding.

**
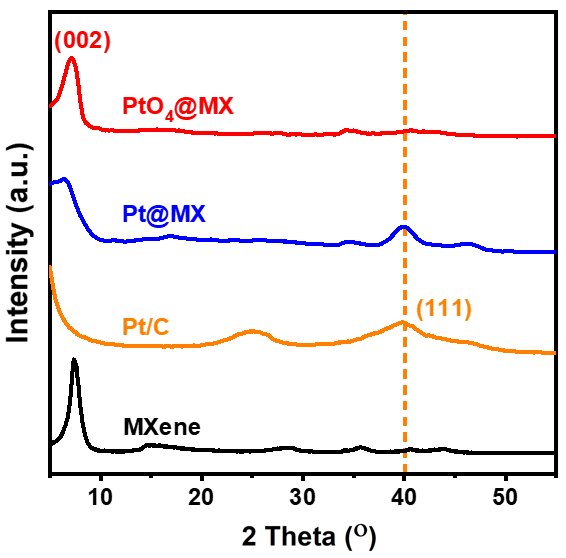
**

**Figure S16.** XRD θ-2θ spectra of pristine MXene, commercial Pt/C, Pt@MX, and PtO_4_@MX.

Bragg's law was employed to calculate the d-spacing between MXene nanosheets before and after electrostatic self-assembly with PtO_4_-DA. The first-order reflection (n = 1), and a wavelength of 1.5406 Å were used for the calculations.


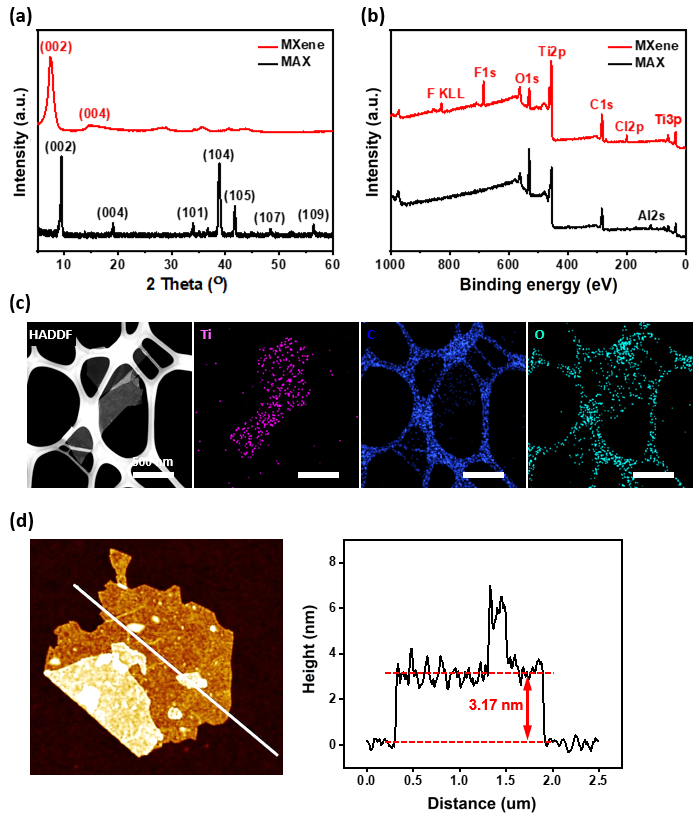


**Figure S17.** Structure characterizations of Ti_3_C_2_T_x_ MXene nanosheets. **(a)** XRD θ-2θ spectra of Ti_3_AlC_2_ MAX powder, and delaminated Ti_3_C_2_T_x_ MXene. **(b)** XPS surveys of MAX powder, and MXene. **(c)** HAADF-STEM image of delaminated MXene nanosheets and the elemental mapping of the corresponding elementals: Ti, C, and O. **(d)** Atomic force microscopy (AFM) image of the MXene nanosheets with the thickness measurement.


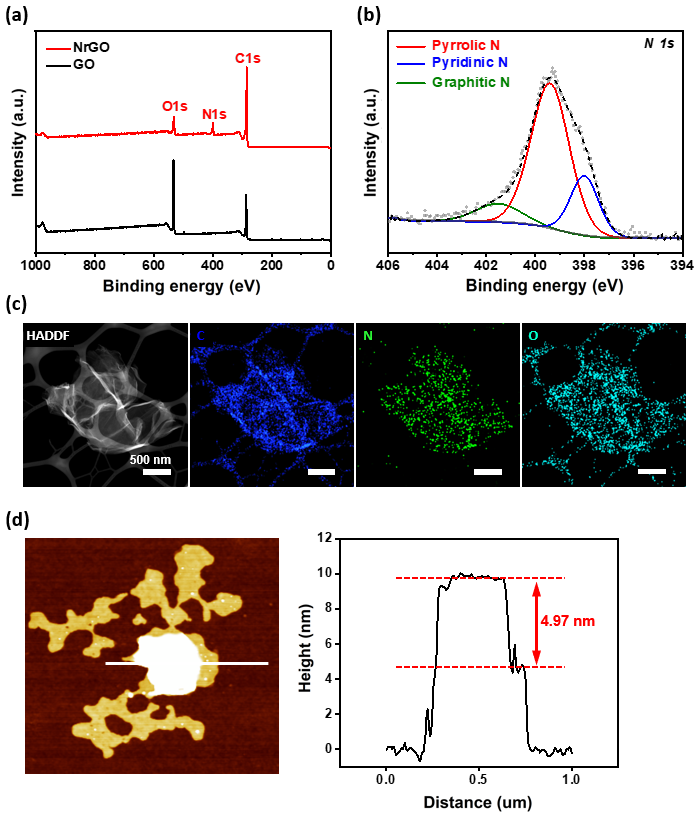


**Figure S18.** Structure characterizations of NrGO nanoflakes. **(a)** XPS surveys of Graphene Oxide (GO), and NrGO. **(b)** XPS N 1s spectrum of NrGO with displaying deconvolution peaks for pyrrolic N, pyridinic N, and graphitic N. **(c)** HAADF-STEM image of NrGO nanoflakes and the elemental mapping of the corresponding elementals: C, N and O. **(d)** AFM image of the NrGO nanoflakes with the thickness measurement.


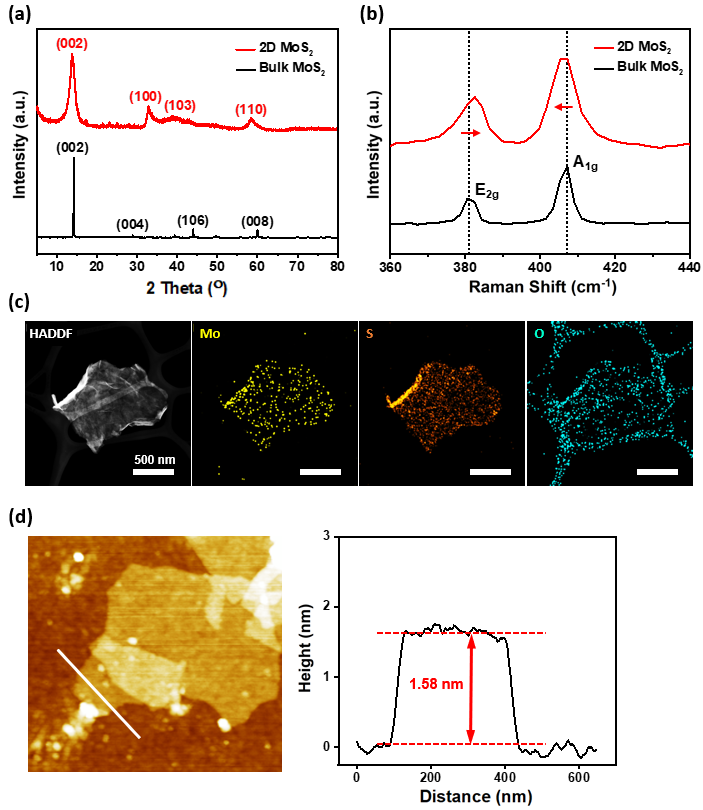


**Figure S19.** Structure characterizations of MoS_2_ nanosheets. **(a)** XRD θ-2θ spectra of bulk MoS_2_, and 2D MoS_2_ nanosheets. **(b)** Raman spectra of bulk MoS_2_, and 2D MoS_2_ nanosheets. **(c)** HAADF-STEM image of delaminated MoS_2_ nanosheets and the elemental mapping of the corresponding elementals: Mo, S, and O. **(d)** AFM image of the MoS_2_ nanosheets with the thickness measurement.


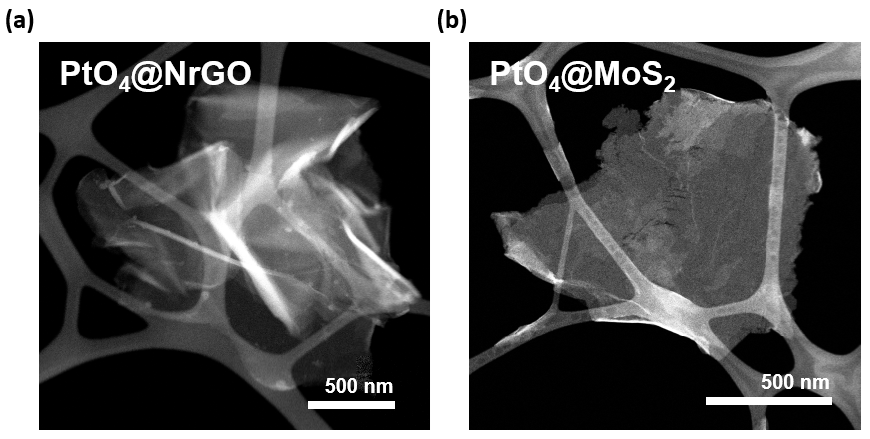


**Figure S20.** HAADF-STEM images of **(a)** PtO_4_@NrGO and **(b)** PtO_4_@MoS_2_ in low magnification.


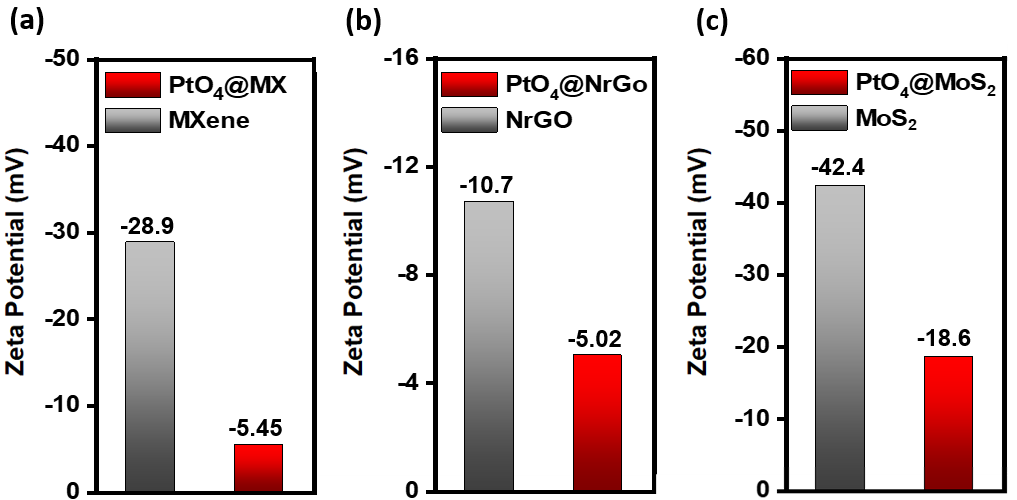


**Figure S21.** Zeta potential measurements of **(a)** PtO_4_@MX, **(b)** PtO_4_@NrGO, and **(c)** PtO_4_@MoS_2_ in the same pH 3.5 condition with those pristine substrates.

**
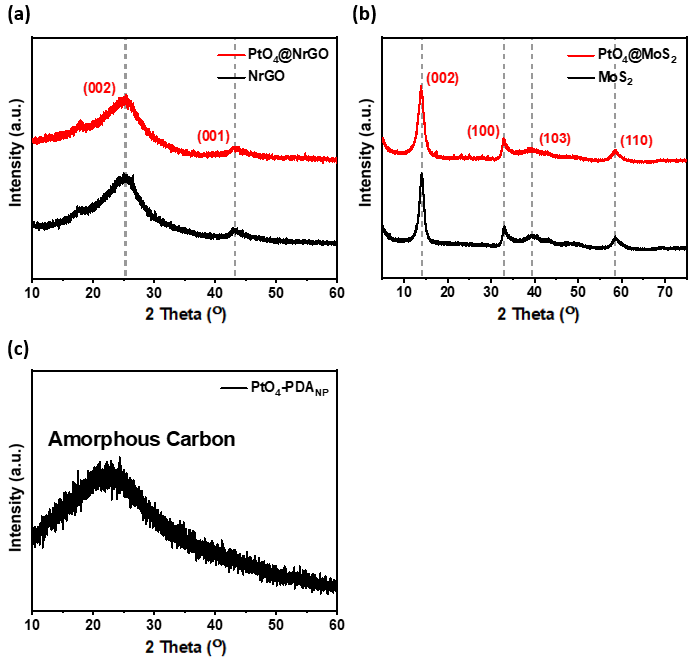
**

**Figure S22.** XRD θ-2θ spectra of **(a)** PtO_4_@NrGO, **(b)** PtO_4_@MoS_2_, and **(c)** PtO_4_-PDA_NP_.


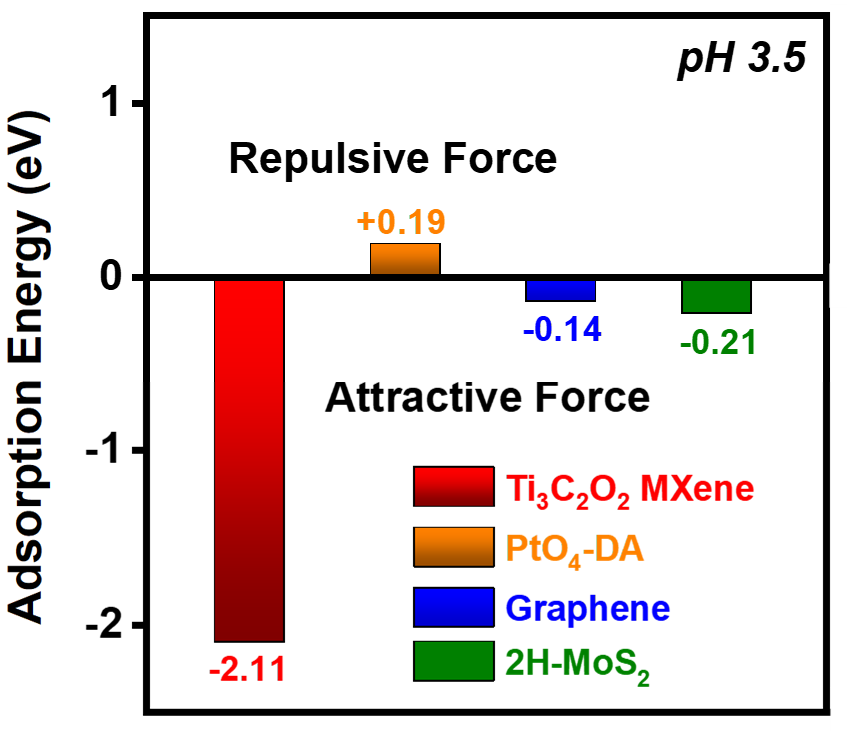


**Figure S23.** DFT calculation for adsorption energy of PtO_4_-DA on Ti_3_C_2_O_2_ MXene, Graphene, 2H-MoS_2_, and PtO_4_-DA.

**
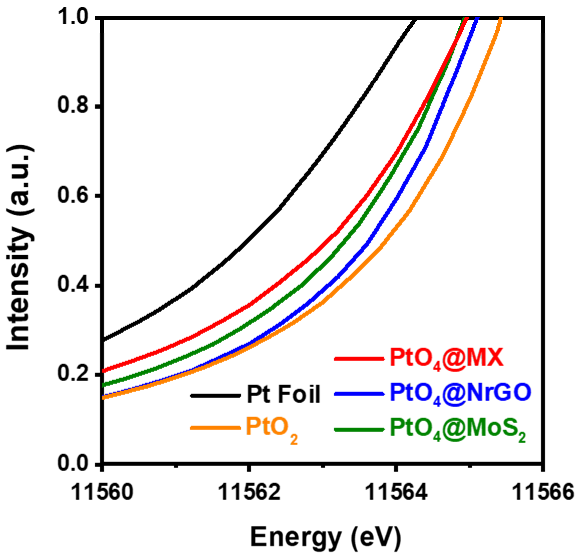
**

**Figure S24.** Magnified XANES spectra at Pt L_3_-edge of PtO_4_@MX, PtO_4_@NrGO, PtO_4_@MoS_2_, PtO_2_, and reference Pt foil.

**
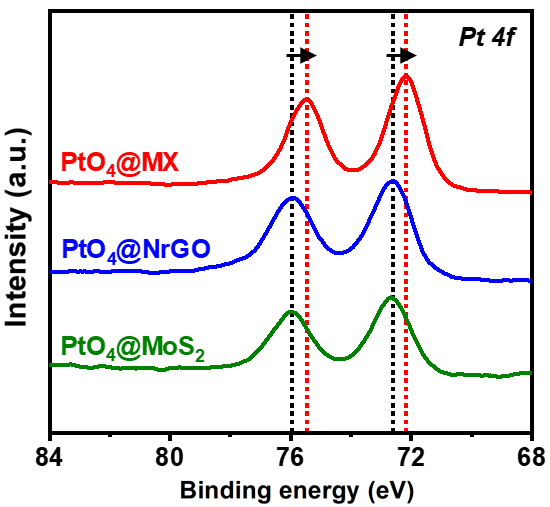
**

**Figure S25.** XPS Pt 4f spectra of PtO_4_@MX, PtO_4_@NrGO, and PtO_4_@MoS_2_.

**
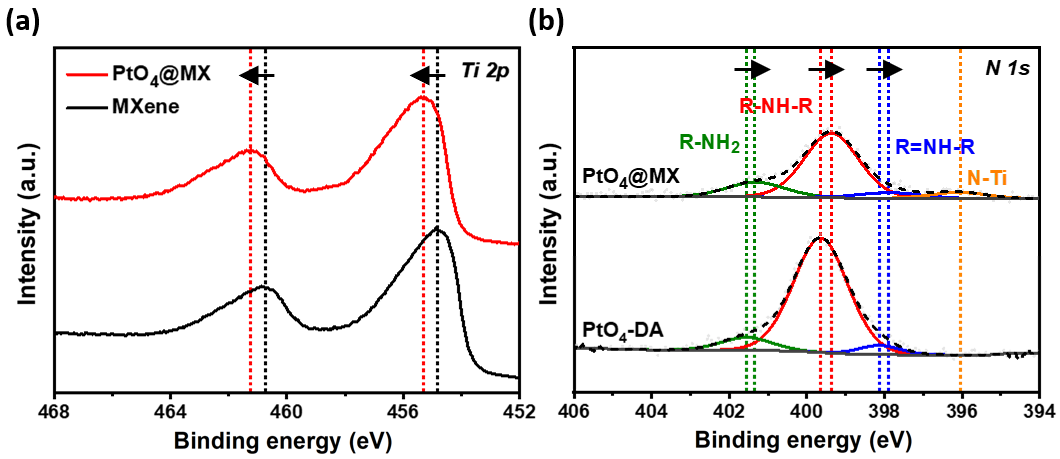
**

**Figure S26. (a)** XPS Ti 2p spectra of MXene before and after PtO_4_ deposition. **(b)** XPS N 1s spectra of PtO_4_-DA before and after electrostatic self-assembly with MXene.

**
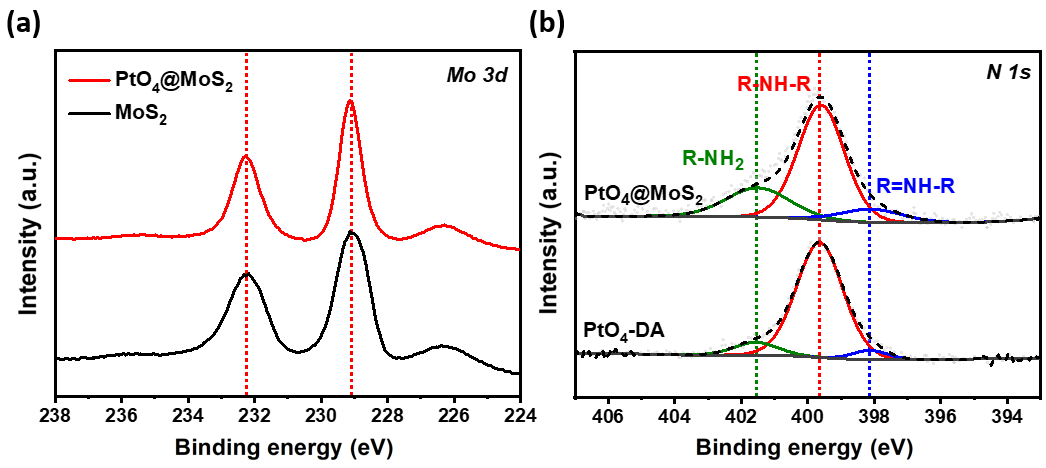
**

**Figure S27. (a)** XPS Mo 3d spectra of MoS_2_ before and after PtO_4_ deposition. **(b)** XPS N 1s spectra of PtO_4_-DA before and after electrostatic self-assembly with MoS_2_.


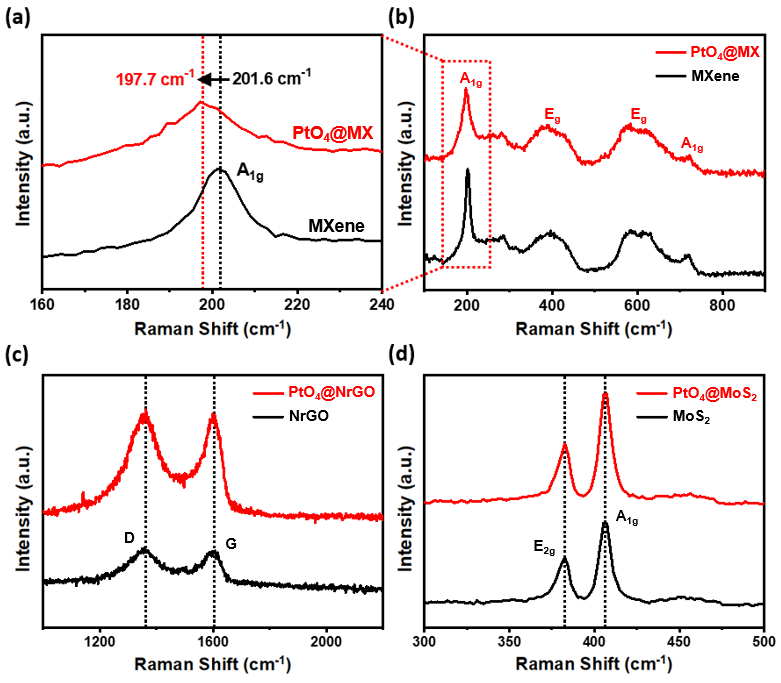


**Figure S28**. Raman spectra of **(a, b)** PtO_4_@MX, **(c)** PtO_4_@NrGO, and **(d)** PtO_4_@MoS_2_ comparing with their pristine substrates.


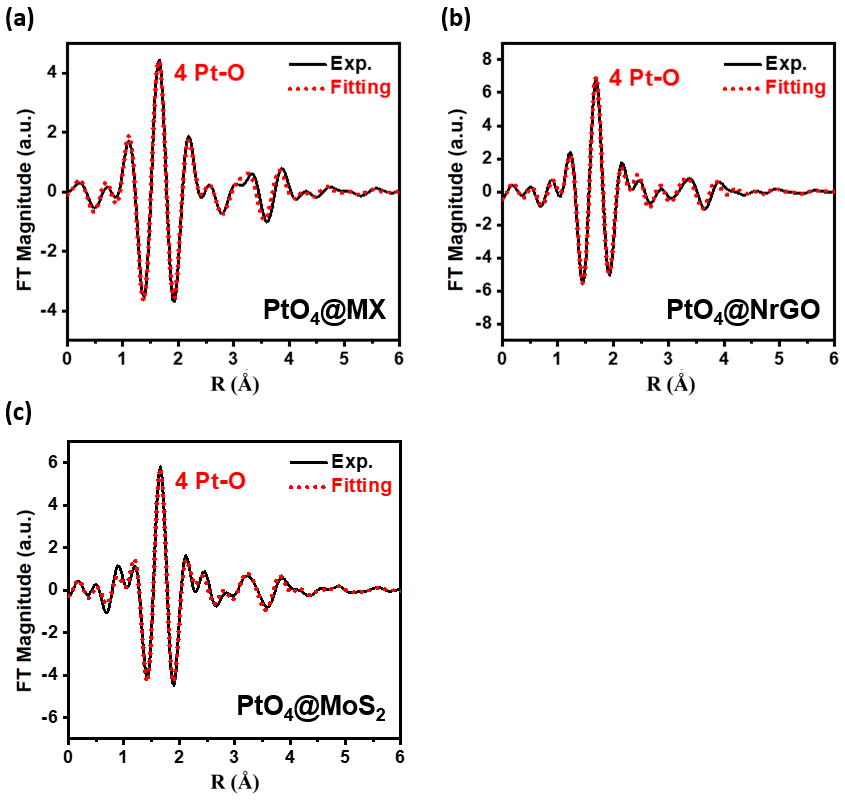


**Figure S29.** Fitted FT-EXAFS spectra of **(a)** PtO_4_@MX, **(b)** PtO_4_@NrGO, and **(c)** PtO_4_@MoS_2_.


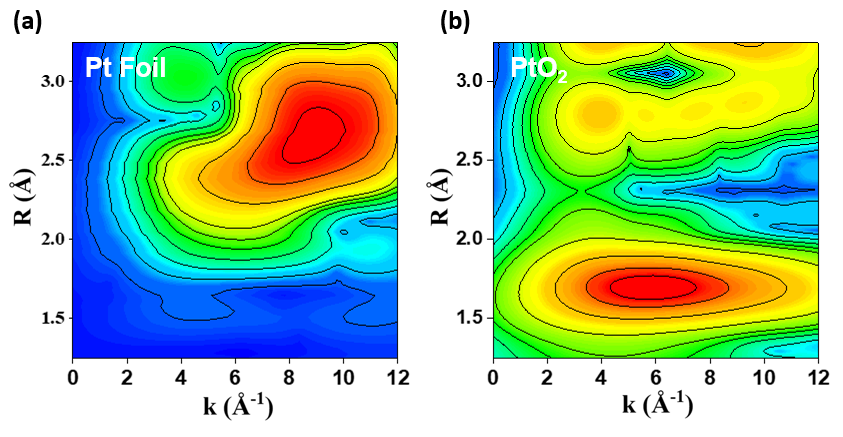


**Figure S30.** WT-EXAFS spectra of **(a)** reference Pt foil, and **(b)** PtO_2_.

**
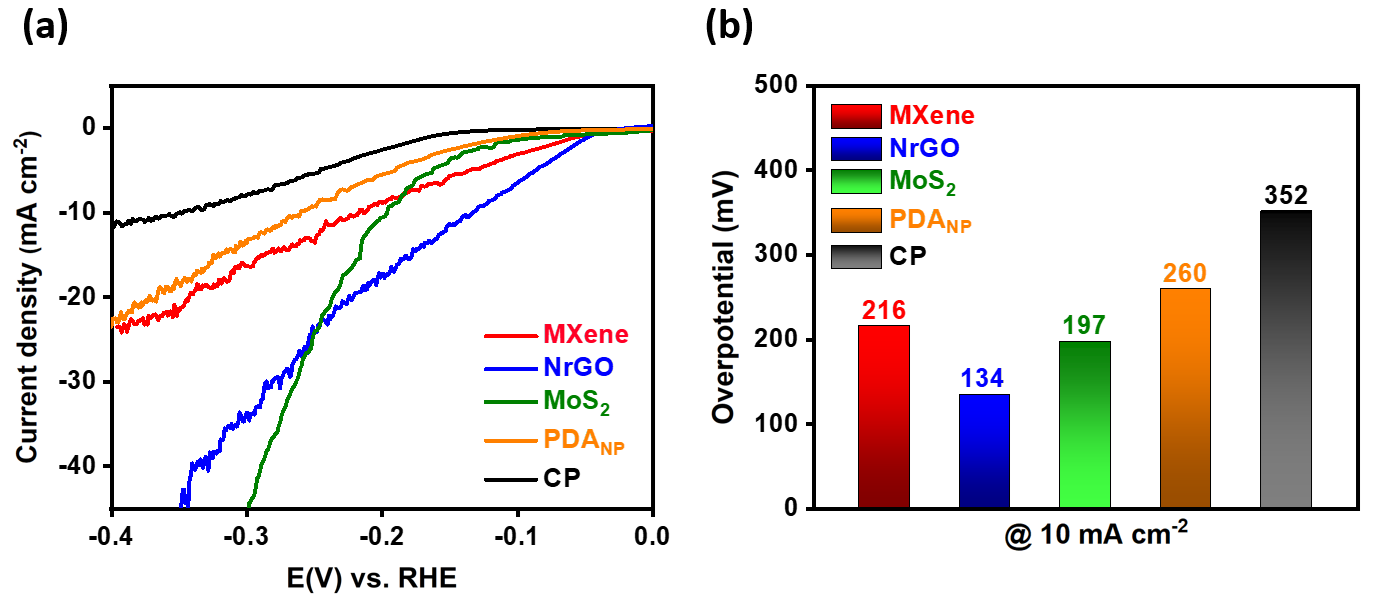
**

**Figure S31. (a)** HER polarization curves of pristine MXene, NrGO, MoS_2_, PDA_NP_, and CP. **(b)** Corresponding overpotential values at the current density of 10 mA cm^-2^.

**
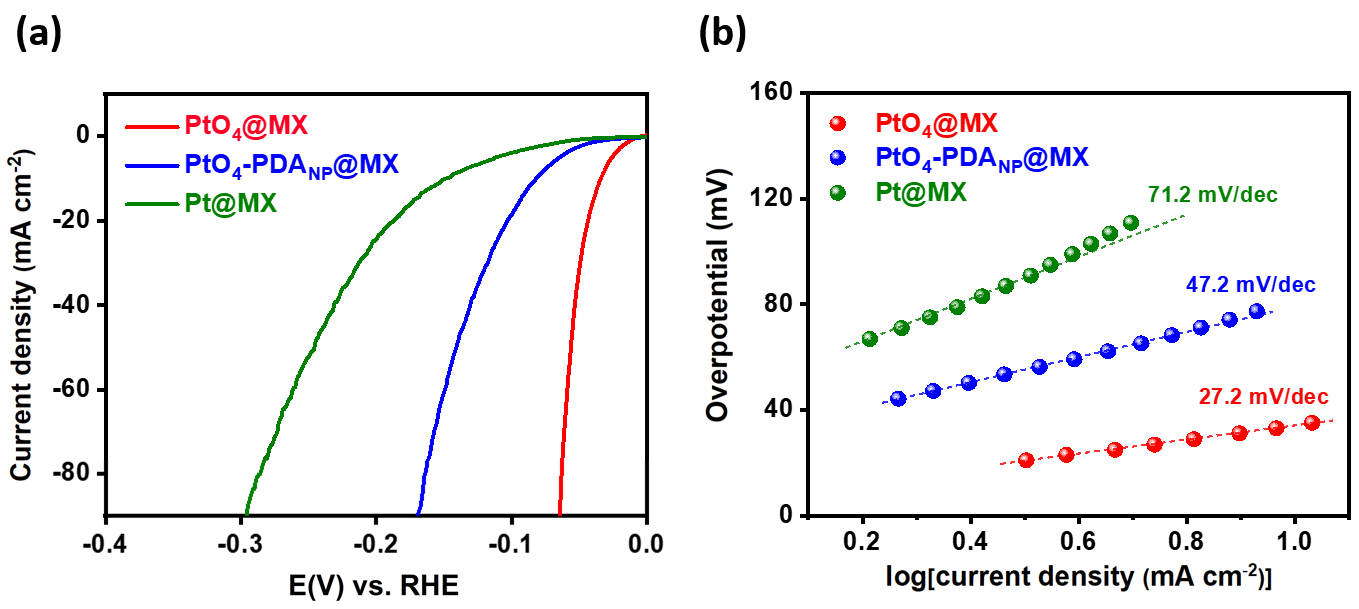
**

**Figure S32. (a)** HER polarization curves of PtO_4_@MX, PtO_4_-PDA_NP_@MX, and Pt@MX. **(b)** Corresponding Tafel slope originated from the LSV curves.

**
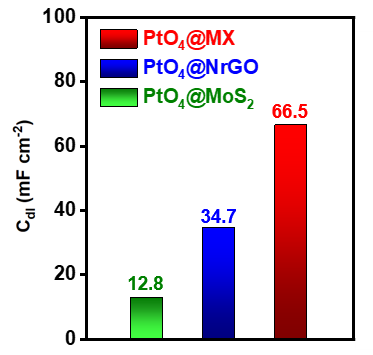
**

**Figure S33.** Double layer capacitance calculated from Cyclic Voltammograms (CV) of PtO_4_@MX, PtO_4_@NrGO, and PtO_4_@MoS_2_.

**
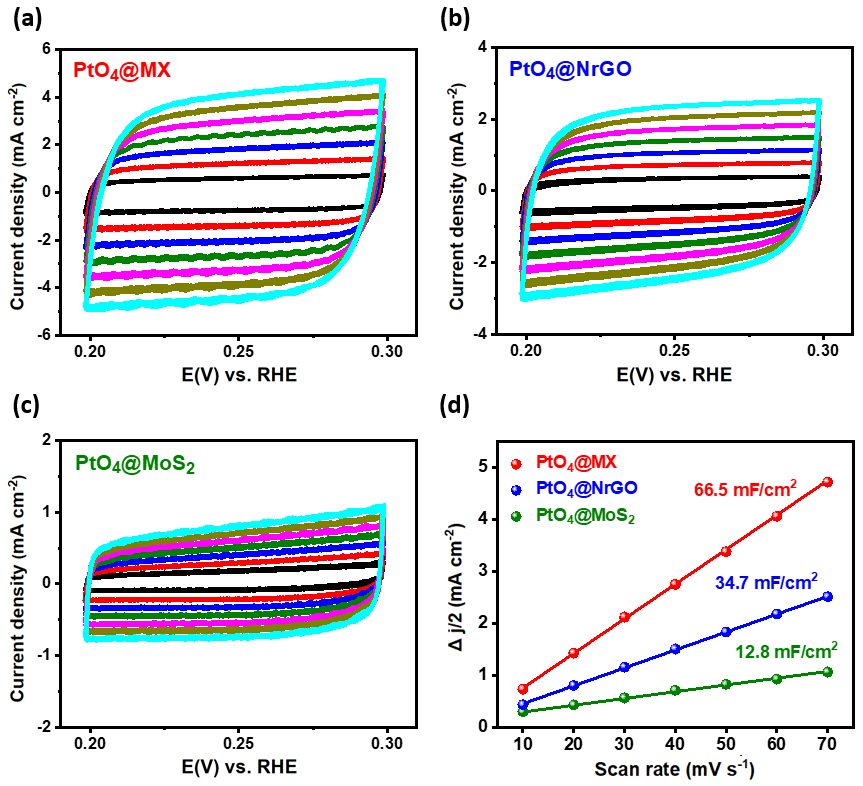
**

**Figure S34.** CV of **(a)** PtO_4_@MX, **(b)** PtO_4_@NrGO, and **(c)** PtO_4_@MoS_2_ at scan rates ranging from 10 to 70 mV s^-1^. **(d)** The corresponding scan rate dependence of the average capacitive currents for PtO_4_@MX, PtO_4_@NrGO, and PtO_4_@MoS_2_.

**
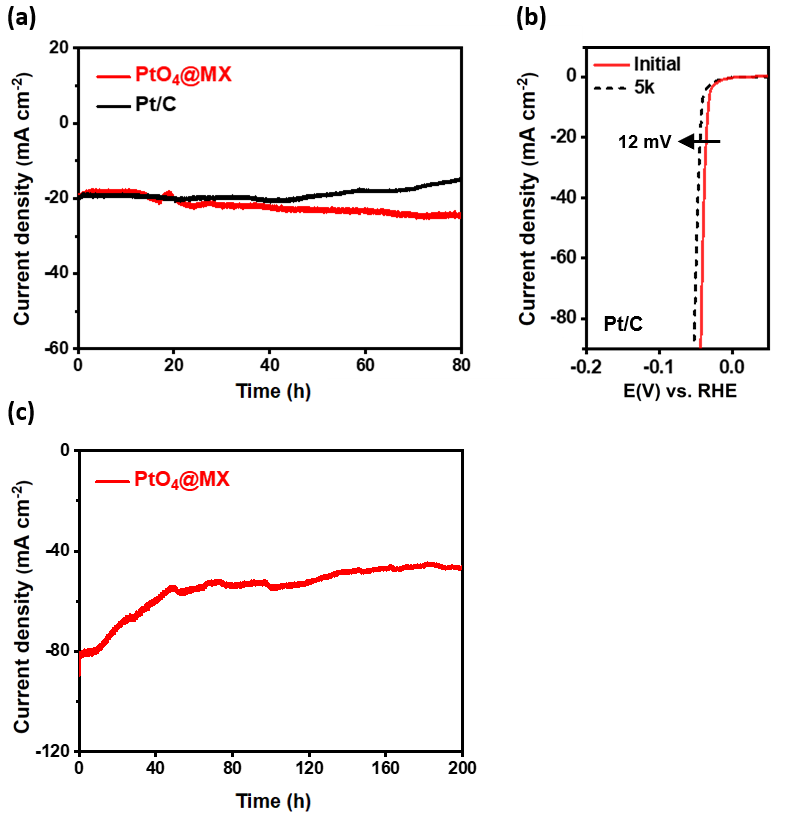
**

**Figure S35. (a)** Chronoamperometry curves of PtO_4_@MX obtained at the overpotential of -40 mV, and 40 wt% commercial Pt/C obtained at the overpotential of -33 mV for 80 hours. **(b)** LSV curves of Pt/C before and after 5,000 LSV cycles at a scan rate of 5 mV s^-1^. (c) Chronoamperometry curves of PtO_4_@MX obtained at the overpotential of -63 mV for 200 hours.


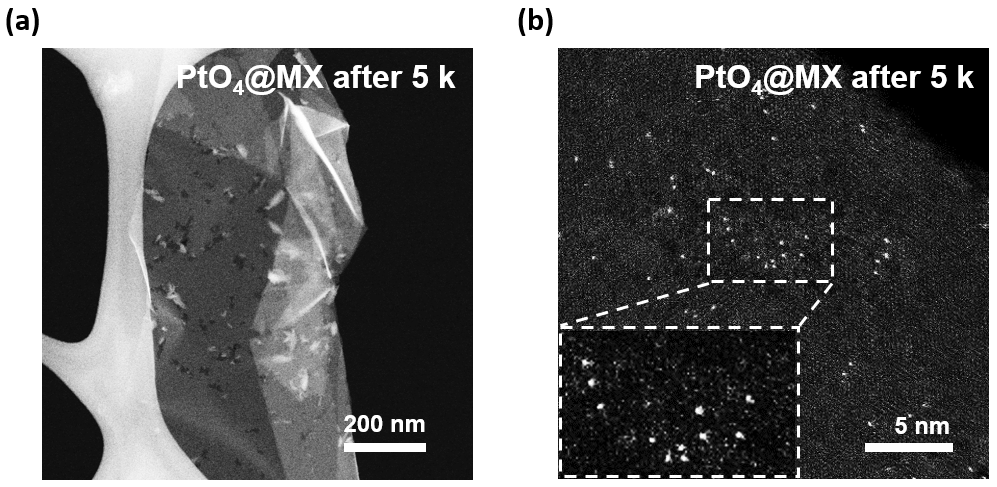


**Figure S36.** HAADF-STEM images of PtO_4_@MX after 5,000 cycle tests in **(a)** low magnification and **(b)** high magnification.

After repeated LSV running for 5,000 cycles, the carbon paper was carefully retrieved and immersed in deionized (DI) water to eliminate any residual acid. Subsequently, it was subjected to low-intensity sonication in 10 mL of DI water for 30 minutes, ensuring the complete dissolution of PtO_4_@MX. Then, PtO_4_@MX was drop-casted onto lacey carbon TEM grids for sampling.

**
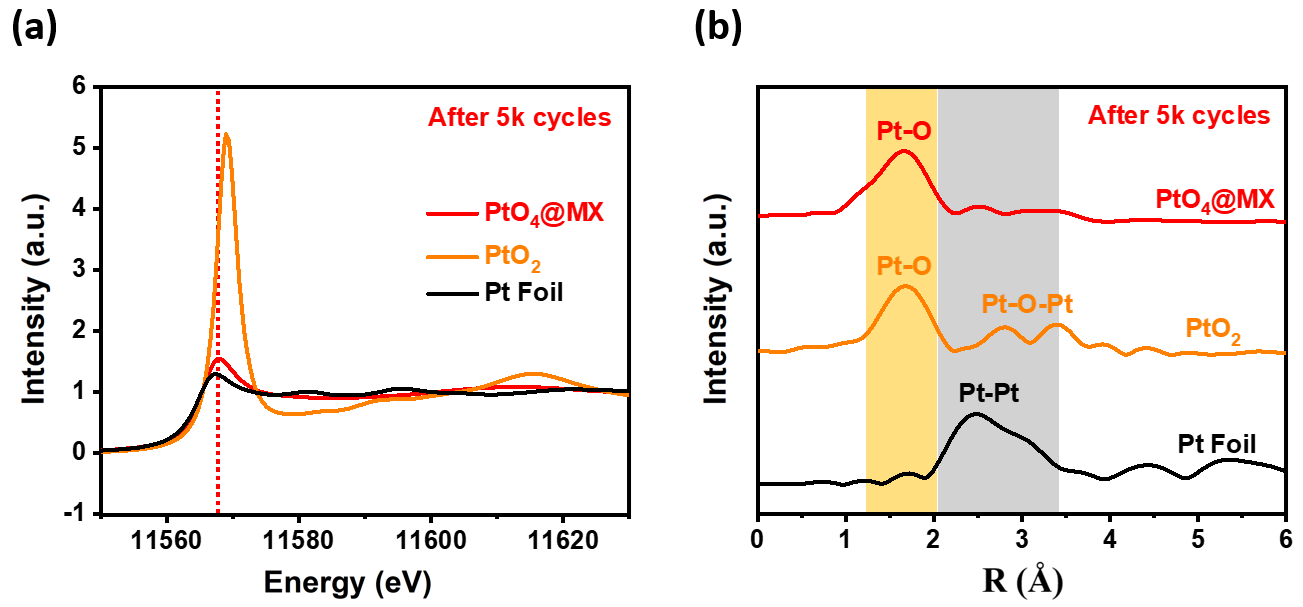
**

**Figure S37. (a)** XANES and **(b)** FT-EXAFS spectra of PtO_4_@MX retrieved after 5,000 LSV cycle tests, with the reference Pt foil and PtO_2_.


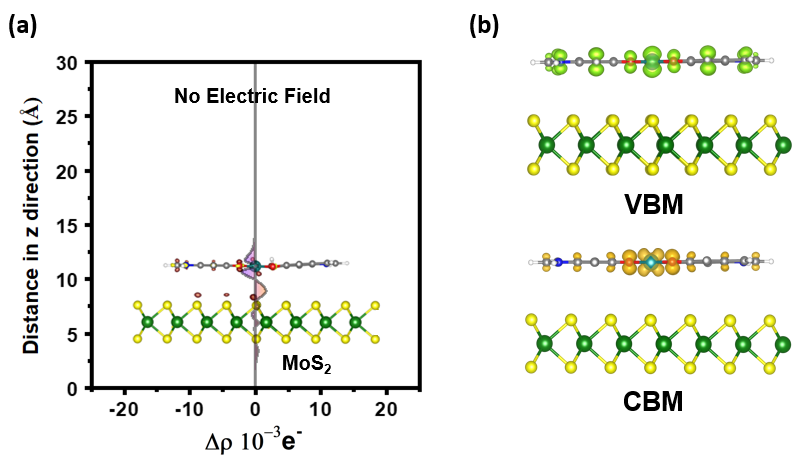


**Figure S38. (a)** Charge difference plot with computational model and localized electric field distribution of PtO_4_@MoS_2_. **(b)** Electron density distribution at the valence band maximum (VBM), and conduction band minimum (CBM) of PtO_4_@MoS_2_ in real space.

Although molybdenum (Mo) and titanium (Ti) are both metallic elements, Ti_3_C_2_O_2_ MXene and 2H-MoS_2_ exhibit fundamentally distinct properties due to their intrinsic electronic structures. Ti_3_C_2_O_2_ MXene demonstrates metallic-like conductivity, attributed to its high charge carrier density and efficient free-electron transport, resulting in exceptionally high electrical conductivity. In contrast, 2H-MoS_2_ possesses a semiconducting nature with an inherent bandgap, necessitating electronic excitation to the conduction band for charge transport, which inherently limits its conductivity. Therefore, it is difficult to facilitate the electron transfer at the hetero-interface between PtO_4_-DA and MoS_2_.

**
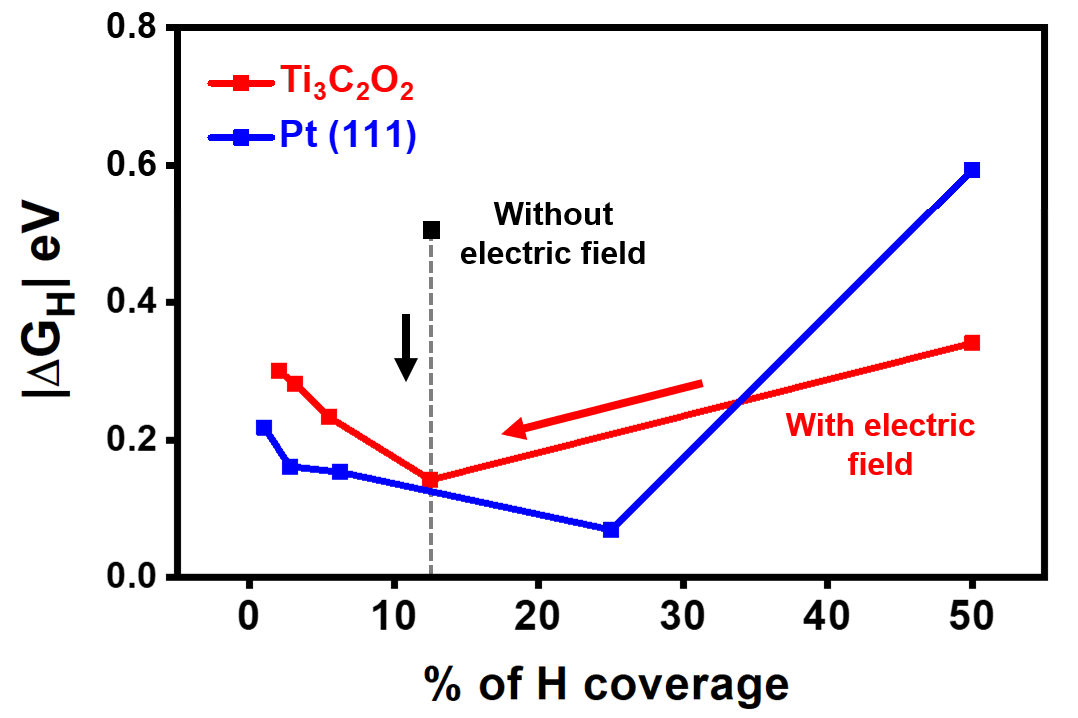
**

**Figure S39.** The variation in Gibbs free energy as a function of hydrogen coverage (%) on Pt (111) and Ti_3_C_2_O_2_ surfaces.

The study employs a computational hydrogen electrode (CHE) model, following DFT-based theoretical methodologies, to examine the dependence of HER activity on surface hydrogen coverage. For Pt (111), the Gibbs free energy approaches a minimal value when the hydrogen coverage reaches 25%, indicating an optimal adsorption condition for HER. Beyond this point, Gibbs free energy increases, suggesting a decrease in catalytic efficiency at higher hydrogen coverage. This trend aligns with existing DFT studies on HER, where Pt (111) surfaces exhibit the most favorable hydrogen adsorption energetics at specific coverage levels. The percentage of hydrogen coverage is calculated using the formula:

$$\% Coverage= \frac{Number of hydrogen atoms \times100}{4\times a\times b}$$

where 𝑎 and 𝑏 define the dimensions of the 𝑎 × 𝑏 supercell, and the factor 4 accounts for the four hollow-top adsorption sites in the unit cell of the Pt (111) slab.

For Ti_3_C_2_O_2_, the hydrogen coverage calculation differs due to the presence of two oxygen-top adsorption sites per unit cell, leading to the following formula:

$$\% Coverage= \frac{Number of hydrogen atoms \times100}{2\times a\times b}$$

The trend observed for Ti_3_C_2_O_2_ differs from that of Pt (111), as its optimal Gibbs free energy occurs at a lower hydrogen coverage. In our self-assembled SAC system, the pre-adsorbed PtO_4_-DA on Ti_3_C_2_O_2_ can regulate the excessive proton adsorption on the substrates, playing a crucial role in modulating the proton coverage. Consequently, the average proton coverage inevitably decreases, approaching the optimal 12.5%, thereby promoting enhanced HER kinetics. This simulation underscores the profound influence of adsorption site variations and structural configurations on HER activity across diverse catalytic surfaces.

**Table S1.** FT-EXAFS fitting results of PtO_4_-DA, PtO_4_@MX, PtO_4_@NrGO, and PtO_4_@MoS_2_.

| **Sample** | **Shell** | **CN** | **R (Å)** | **σ^2^** | **ΔE_0_ (eV)** | **R factor** |
| --- | --- | --- | --- | --- | --- | --- |
| **PtO_4_-DA** | Pt-O | 4 | 2.0573 | 0.0028 | 12.750 | 0.0023 |
| **PtO_4_@MX** | Pt-O | 4 | 2.0029 | 0.0032 | 14.870 | 0.0054 |
| **PtO_4_@NrGO** | Pt-O | 4 | 2.0408 | 0.0021 | 14.158 | 0.0057 |
| **PtO_4_@MoS_2_** | Pt-O | 4 | 2.0236 | 0.0036 | 16.584 | 0.0078 |

CN: coordination numbers of identical atoms surround the metal atom, $R$: interatomic distance between atoms, σ^2^: Debye-Waller factors, ΔE_0_: energy shift, and R factor: goodness of fit. Ѕ_0_^2^ was fixed at 0.77 for all samples. This value is calculated from the experimental EXAFS fit of reference Pt foil conducted in the same operation, by fixing CN as the known crystallographic value.

**Table S2.** Weight percentage of Pt in all samples measured by ICP-OES analysis.

| **PtO_4_-PDA_NP_** | **PtO_4_@MX** | **PtO_4_@NrGO** | **PtO_4_@MoS_2_** |
| --- | --- | --- | --- |
| **4.5 wt%** | **1.4 wt%** | **4.9 wt%** | **3.6 wt%** |
| **PtO_4_-PDA_NP_@MX** | **Pt@MX** |  |  |
| **2.9 wt%** | **5.1 wt%** |  |  |

**Table S3.** Comparison of HER activity performances of PtO_4_@MX and the reported electrocatalysts based on various 2D nanomaterials in 0.5 M H_2_SO_4_.

| **Electrocatalyst** | **Substrate** | **Overpotential at 10 mA cm^-2^ / mV** | **Tafel Slope / mV dec^-1^** | **Reference** |
| --- | --- | --- | --- | --- |
| **PtO_4_@MX** | **Carbon paper** | **33.1** | **27.2** | **This work** |
| **PtO_4_@NrGO** | **Carbon paper** | **42.6** | **34.7** | **This work** |
| **PtO_4_@MoS_2_** | **Carbon paper** | **55.8** | **37.9** | **This work** |
| **Ti_3_C_2_T_x_-Pt_SA_** | **Glassy carbon electrode** | **38.0** | **45.0** | *Nano. Lett.* **2022**, 22, 1398-1405 |
| **Pt SA-PNPM** | **Glassy carbon electrode** | **35.0** | **31.0** | *ACS Nano* **2022**, 16, 4116-4125 |
| **Pt SA-PNM** | **Glassy carbon electrode** | **57.0** | **55.0** | *ACS Nano* **2022**, 16, 4116-4125 |
| **SA-Pt/MoS_2_** | **Carbon paper** | **44.0** | **34.8** | *Small* **2022**, 18, 2104824 |
| **MXene@Pt/SWCNT** | **Filtrated film** | **62.0** | **78.0** | *Adv. Funct. Mater.* **2020**, 30, 2000693 |
| **40Pt-TBA-Ti_3_C_2_T_x_** | **Glassy carbon electrode** | **67.8** | **69.8** | *Ind. Eng. Chem. Res*. **2020**, 59, 1822-1828 |
| **Pt SA-MoS_2_** | **Glassy carbon electrode** | **59.0** | **31** | *Nat. Commun.* **2020**, 11, 4558 |
| **Pt NCs-MXene** | **Carbon paper** | **40.0** | **50.8** | *J. Electrochem. Soc.* **2021**, 168, 096504 |
| **Mo_2_TiC_2_T_x_-Pt_SA_** | **Carbon paper** | **30.0** | **30.0** | *Nat. Catal.* **2018**, 3, 773-782 |
| **ALD Pt/NGN** | **Rotating disk electrode** | **-** | **29.0** | *Nat. Commun.*  **2019**, 7, 13638 |

**Table S4.** R_s_ and R_ct_ values of PtO_4_-DA, PtO_4_@MX, PtO_4_@NrGO, and PtO_4_@MoS_2_ in EIS Nyquist plots.

| **Sample** | **Series Resistance (R_s_) / Ohm** | **Charge Transfer Resistance (R_ct_) / Ohm** |
| --- | --- | --- |
| **PtO_4_@MX** | 1.0845 | 0.13579 |
| **PtO_4_@NrGO** | 1.1857 | 0.17074 |
| **PtO_4_@MoS_2_** | 1.3060 | 0.18409 |
| **PtO_4_-PDA_NP_** | 1.5275 | 0.26885 |

**Table S5.** Calculated Fermi level of Ti_3_C_2_ MXene with different functional groups, and our PtO_4_@Ti_3_C_2_O_2_ model.

| **Model** | **Calculated Fermi Level (eV)** |
| --- | --- |
| **Ti_3_C_2_ (pristine)** | -3.735 eV |
| **Ti_3_C_2_O_2_ (O-functionalized)** | -5.957 eV |
| **Ti_3_C_2_(OH)_2_ (OH-functionalized)** | -0.370 eV |
| **PtO_4_@Ti_3_C_2_O_2_** | -5.518 eV |
| **PtO_4_ (isolated)** | -4.223 eV |

The work function (Φ) was determined as the difference between the vacuum level (set to 0.0 eV) and the Fermi energy of the material. This allowed for a consistent comparison of Fermi levels across different materials in the heterostructure. These results demonstrate that the Fermi energy of Ti_3_C_2_ can be modulated significantly (from -0.370 to -5.957 eV), depending on the nature of the surface terminations.

Although interfacial energy level alignment plays a pivotal role in facilitating electron transfer, accurately predicting the exact ratio of surface termination groups in experimental MXene samples remains highly challenging due to their sensitivity to synthesis conditions and post-treatment environments. To conservatively address this uncertainty, we employed a Ti_3_C_2_O_2_ model—which possesses the highest work function among the commonly reported terminations—to demonstrate that efficient electron transfer can still occur even under the least favorable conditions. In realistic systems, where mixed terminations such as –O and –OH coexist, the overall work function is expected to be significantly lower, thereby further enhancing interfacial electron transfer beyond that observed in the –O-only model.

**Table S6.** Average Bader charge calculation on the elements of Ti_3_C_2_O_2_ MXene with and without PtO_4_.

| **Element** | **Without PtO_4_** | **With PtO_4_ on Top** |
| --- | --- | --- |
| **Ti** | 10.150 | 10.152 |
| **C** | 5.662 | 5.666 |
| **O-top** | 7.113 | 7.132 |
| **O-bottom** | 7.113 | 7.166 |
